# HLA class I molecular variation and peptide-binding properties suggest a model of joint divergent asymmetric selection

**DOI:** 10.1007/s00251-016-0918-x

**Published:** 2016-05-27

**Authors:** Stéphane Buhler, José Manuel Nunes, Alicia Sanchez-Mazas

**Affiliations:** 1 Laboratory of Anthropology, Genetics and Peopling History, Department of Genetics and Evolution, Anthropology Unit, University of Geneva, Geneva, Switzerland; 2 Transplantation Immunology Unit & National Reference Laboratory for Histocompatibility, Department of Genetic and Laboratory Medicine, Geneva University Hospital, Geneva, Switzerland; 3 Institute of Genetics and Genomics in Geneva (iGE3), University of Geneva, Geneva, Switzerland

**Keywords:** HLA class I polymorphism, Functional variation, Peptide-binding properties, Asymmetric balancing selection, Heterozygous advantage, Immune protection

## Abstract

The main function of HLA class I molecules is to present pathogen-derived peptides to cytotoxic T lymphocytes. This function is assumed to drive the maintenance of an extraordinary amount of polymorphism at each HLA locus, providing an immune advantage to heterozygote individuals capable to present larger repertories of peptides than homozygotes. This seems contradictory, however, with a reduced diversity at individual HLA loci exhibited by some isolated populations. This study shows that the level of functional diversity predicted for the two HLA-A and HLA-B genes considered simultaneously is similar (almost invariant) between 46 human populations, even when a reduced diversity exists at each locus. We thus propose that HLA-A and HLA-B evolved through a model of joint divergent asymmetric selection conferring all populations an equivalent immune potential. The distinct pattern observed for HLA-C is explained by its functional evolution towards killer cell immunoglobulin-like receptor (KIR) activity regulation rather than peptide presentation.

**Electronic supplementary material:**

The online version of this article (doi:10.1007/s00251-016-0918-x) contains supplementary material, which is available to authorized users.

## Introduction

Located in the 6p21.3 chromosomal region, HLA class I genes are among the most polymorphic of the human genome (Robinson et al. 2015; The MHC sequencing consortium 1999). To some exceptions (e.g., erythrocytes), class I molecules are expressed ubiquitously by every cellular type of the body. Their main function is to present small antigenic peptides (mostly nonamers) of intracellular origin to the receptors of CD8+ cytotoxic T lymphocytes (TCR). During an infection by a pathogen (e.g., a virus), infected cells generally increase their membrane expression of class I molecules in order to be tagged for destruction by the adaptive immune system (Parham 2009).

The peptide-binding region (PBR) of HLA class I molecules, which presents the antigenic peptides, is encoded by exons 2 and 3 of the corresponding genes, where most of the polymorphism is observed (Little and Parham 1999). For this reason, the huge HLA class I diversity observed in human populations, now represented by more than ten thousands of different alleles (10,297 according to release 3.22.0 of the IMGT/HLA database (Robinson et al. 2015)) showing variable frequencies worldwide (Sanchez-Mazas et al. 2011, 2013; Santos et al. 2015), is generally thought to be functionally relevant and maintained by different forms of balancing selection (Di et al. 2015; Garrigan and Hedrick 2003; Meyer et al. 2006; Meyer and Thomson 2001; Solberg et al. 2008; Spurgin and Richardson 2010). Although allele frequency-dependent selection (Slade and McCallum 1992; Takahata and Nei 1990) and selection fluctuating in space and time (Hedrick 2002) may also be at work, the mechanism that is most often invoked to explain this huge diversity is heterozygote advantage (overdominance). This suggests that heterozygote individuals carrying different alleles at a given locus are able to present a larger range of antigenic peptides than homozygotes as HLA genes are co-dominantly expressed (Doherty and Zinkernagel 1975; McClelland et al. 2003; Penn et al. 2002; Thursz et al. 1997). Such individuals are, in this way, better protected against pathogens (Prugnolle et al. 2005; Qutob et al. 2011; Sanchez-Mazas et al. 2012). Taking into account the remarkable sequence variation observed within the PBR (Bronson et al. 2013; Buhler and Sanchez-Mazas 2011), some studies support the divergent allele advantage (DAA) hypothesis which assumes both asymmetric heterozygote advantage and high divergence of allele sequences. According to DAA (Wakeland et al. 1990), heterozygotes carrying divergent alleles would have an enhanced ability to present larger sets of peptides than heterozygotes carrying molecularly close alleles. On the other hand, based on the numerous population studies that have been performed during the last decades (Buhler and Sanchez-Mazas 2011; Nunes et al. 2010; Riccio et al. 2013; Sanchez-Mazas et al. 2011; Solberg et al. 2008), one remarkable observation, which appears contradictory with the abovementioned hypotheses, is that large proportions of homozygotes at one or more loci are often found in small-sized and isolated populations such as Amerindians, Taiwanese Aborigines, and Pacific islanders, as a likely result of rapid genetic drift during their migrations’ history. In this context, the genetic homogeneity of Amerindians has been related to their high rate of mortality following European colonization, which would have rendered them particularly susceptible to newly introduced diseases (Black 1992). However, a very contrasting situation is observed in some Aboriginal populations from Taiwan, which are numerically very large despite being among the most homogeneous populations in the world according to HLA (Lin et al. 2000). Indeed, recent demographic data on the Ami and Paiwan indicate census of 200,604 and 96,334 individuals, respectively, whereas the Ami and the Paiwan exhibit a homozygozity of 43 and 76 % at HLA-A with one single allele (A*24:02) reaching a frequency of 64 and 86.3 %, respectively. Such numbers are not consistent with these tribes going extinct. Thus, we may wonder whether additional mechanisms other than heterozygote advantage are involved to ensure a sufficient immune protection in these populations. Looking at other species, a decreased survival has been associated to MHC homozygosity by some studies (Froeschke and Sommer 2005; Huchard et al. 2010; Worley et al. 2010), but not by others (Ilmonen et al. 2007; Mainguy et al. 2006); this also leaves open the possibility of additional selective forces at play.

One attractive approach to tackle this question is to investigate the patterns of HLA genetic variation observed at the population level in relation to the functionality of the HLA molecules and in particular to their peptide-binding specificities. The identification of residues involved in peptide binding was first assessed by crystallographic studies determining the three-dimensional structure of the HLA-A2 molecule (Bjorkman et al. 1987; Saper et al. 1991). They were followed by deeper investigations on class I molecules properties (Chelvanayagam 1996; Kangueane et al. 2001; Reche and Reinherz 2003), further leading to the definition of class I supertypes (i.e., groups of alleles sharing chemical properties at the B and F pocket-like structures of the PBR) (Francisco et al. 2015; Sidney et al. 2008). As a crucial question was to characterize both MHC ligands and peptide motifs involved in peptide binding, diverse database resources like MHCPEP (Brusic et al. 1998), SYFPEITHI (Rammensee et al. 1999), and IEDB (Peters et al. 2005; Vita et al. 2010) were also created. In addition, because the very high level of polymorphism observed in the PBR makes the description of all possible HLA-peptide combinations very challenging, several computer methods taking into account the molecular information of both the PBR and the presented peptides were developed to predict peptide binding even in the absence of experimental data (Hoof et al. 2008; Liao and Arthur 2011a; Lundegaard et al. 2008; Lundegaard et al. 2010; Rapin et al. 2001; Roomp et al. 2010; Thomsen et al. 2013 and Liao and Arthur 2011b for a review). Such developments, together with our recent study questioning the functional relevance of HLA class I supertypes (Francisco et al. 2015), strongly motivated us for investigating in more depth the relationship between the HLA polymorphism and its immune function in a molecular evolutionary framework.

In this study, we thus combine the use of the peptide-binding prediction tools described above (in particular MHCcluster 2.0; Thomsen et al. 2013) with an extensive analysis of molecular diversity in a large set of HLA-typed population samples (6094 individuals from 46 populations worldwide, all of them tested at the second field level of resolution for three class I loci HLA-A, HLA-B, and HLA-C) to explore the functional relevance of the HLA class I polymorphism in human populations. By analyzing the nucleotide diversity at exons 2 and 3 of the three classical HLA class I genes, we first explore the distribution and level of variability of the amino acid residues in the PBR in relation to their involvement in peptide binding in order to reassert previous observations and analyses made on class I genes (Hedrick et al. 1991; Yang et al. 2005). Based on these confirmatory results, we then estimate pairwise molecular distances and predict pairwise peptide-binding distances between all alleles and corresponding molecules observed in our dataset and we use them to assess whether different populations exhibit similar amounts of molecular divergence and peptide-binding coverage, both at individual class I loci and by considering groups of loci together. By using this original approach taking into account the putative immune potential of different populations, our aim is to identify on a thorough statistical basis whether and how balancing selection—and more particularly DAA—may explain the evolution of the HLA class I polymorphism in all populations despite heterogeneous levels of HLA diversity at individual loci due to contrasted demographic histories.

## Material and methods

### Population data

Population samples typed simultaneously at the three classical class I loci HLA-A, HLA-B, and HLA-C were taken from the Gene[VA] database (Nunes et al. 2014). A total of 6094 individuals from 46 populations were retained after filtering the data (e.g., for sufficient sample size, adequate level of typing resolution, and more). A summary of the population data is given in Table 1 and the details (including the filtering criteria) in Supplementary Material and Methods (Online Resource 1). Two criteria were used to categorize the populations, (1) their geographic location in different continental (sub)regions, Europe (EUR), North Africa (NAFR), Sub-Saharan Africa (SAFR), West Asia (WASI), Northeast Asia (NEASI), North America (NAME), South America (SAME), Southeast Asia (SEASI), and Oceania (OCE), and (2) their assumed demographic history through either rapid genetic drift (RGD), for small-sized and isolated populations, or slow genetic drift (SGD) for the others (large outbred populations).

**Table 1:** Summary of the population data

Region	Npop (RGD/SGD)	*N*	Mean sample size^a^
EUR	5 (0/5)	1563	312.6 (±775.25)
NAFR	1 (0/1)	230	230 (NA)
NAME	1 (1/0)	149	149 (NA)
NEASI	2 (0/2)	356	178 (±36.77)
OCE	4 (4/0)	399	99.75 (±123.96)
SAFR	7 (0/7)	1225	175 (±134.3)
SAME	2 (2/0)	212	106 (±90.51)
SEASI^b^	20 (12/8)	1637	81.85 (±103.98)
WASI	4 (0/4)	323	80.75 (±49.33)
	46 (19/27)	6094	

*Npop* number of population samples, *SGD* slow genetic drift, *RGD* rapid genetic drift, *N* number of individuals, *NA* not available, *EUR* Europe, *NAFR* Northern Africa, *NAME* Northern America, *NEASI* Northeastern Asia, *OCE* Oceania, *SAFR* Sub-Saharan Africa, *SAME* Southern America, *SEASI* Southeastern Asia, *WASI* Western Asia

^a^Mean sample size (±2*standard deviation)

^b^Including 15 populations from Taiwan

### Statistical analyses

#### Characterizing the HLA class I molecular diversity at the peptide-binding regions

Exon 2 and 3 sequence alignments of the three HLA class I genes A, B, and C were downloaded from the IMGT/HLA database and pre-formatted as described in Supplementary Material and Methods (Online Resource 1). These were used to calculate pairwise molecular distances (PMD) among the 328 HLA class I alleles (86, 179, and 63 alleles at HLA-A, HLA-B, and HLA-C, respectively) observed in the 46 available population samples, estimated with Arlequin 3.11 (Excoffier and Lischer 2010) by counting the number of nucleotide differences between their corresponding sequences. Shannon entropy (Shannon 1948) is a very sensitive measure of diversity widely used in biology for estimating the variability of sequence data, including HLA (Reche and Reinherz 2003), that also allows to distinguish sequence variability from heterozygosity-based population diversity analyses. To relate the molecular diversity (at exons 2 and 3) of HLA alleles to the peptide-binding properties of their corresponding HLA molecules (defined by amino acid changes in the PBR), all 183 codons of these two regions were characterized by several criteria: (i) the maximal value of entropy was estimated for each codon (*H*_CODON_MAX_ hereafter, details in Supplementary Material and Methods, Online Resource 1), allowing to categorize all codons as containing at least one non-synonymous site (label NS), only synonymous site(s) (label S), or as being monomorphic (label M); (ii) the codons were also classified as coding or not coding for the residues forming the six pocket-like structures (A, B, C, D, E, and F) of the PBR as defined by the crystallographic study of Saper et al. (1991). These pockets accommodate the amino acid residues of the antigenic peptides presented by the HLA molecules (note, however, that the central C, D, and E pockets were regrouped into a single CDE pocket for the analyses, see Supplementary Material and Methods, Online Resource 1). As a result, 34 codons were labelled P (for pocket) and 149 NP (for non-pocket); (iii) finally, a broader definition of peptide-binding residues taking into account 31 additional codons (Supplementary Material and Methods, Online Resource 1) was used to classify the codons into two additional categories, binding (B) and non-binding (NB); of the 183 codons, 65 were labelled B and 118 NB.

We then checked whether polymorphic (NS and S) and non-polymorphic (M) codons were randomly distributed within the PBR, when categorized as coding or not for residues forming the pocket-like structures (P or NP), and as coding or not for residues involved in peptide binding (B or NB). To that aim, we assessed the independence between the level of polymorphism of the codons and their involvement in pocket-like structures or peptide binding by using Pearson’s chi-squared tests, and we estimated their respective contribution to a significant relationship, in case of rejection, by standardized residuals (stdres) (Agresti 2007). The distribution of *H*_CODON_MAX_ values was also used to compare more precisely the categories of codons (P, NP, B, and NB) with Wilcoxon-Mann-Whitney rank sum tests and box-and-whisker plots. We also used a linear model followed by a post hoc Tukey’s HSD test in order to compare the *H*_CODON_MAX_ values at a more detailed structural level using the residues of the four individual pockets A, B, CDE, and F and the non-pocket (NP) residues. All the analyses described above were performed on the three HLA class I loci (A, B, and C) considered separately but also on the three loci taken together (ABC) by using combined sequence alignments.

#### Predicting peptide-binding distances between HLA class I molecules

The method MHCcluster 2.0 (Thomsen et al. 2013), recently developed to cluster HLA molecules according to their peptide-binding specificities, was applied to estimate pairwise peptide-binding distances (PPBD) between the 328 class I variants considered in this study. For a given HLA molecule, MHCcluster 2.0 predicts the binding of its corresponding PBR to a set of 50,000 predefined natural peptides by using the NetMHCpan method (Hoof et al. 2008). Next, the correlation between the top 10 % strongest peptides bound by different molecules is used to measure a peptide-binding similarity between them. This similarity is +1 if the PBRs of two different molecules have a perfect binding specificity overlap (i.e., both PBRs bind exactly to the same top 10 % peptides) and −1 if the two PBRs share no specificity overlap. Given this similarity, the peptide-binding distance between two molecules is defined as (1-similarity). These distances are then transformed to fall within the range [0–1] by dividing them by the largest distance in the dataset analyzed (Morten Nielsen, personal communication).

#### Estimating the mean increase of molecular distance and the mean gain in peptide-binding coverage in different populations

We first investigated the putative relationship between the numbers of HLA alleles (*k*) observed in the populations and the depth of their immune repertoire. Indeed, under DAA selection, one may expect that alleles found in small-sized and isolated populations are increasingly divergent in order to ensure a sufficient immune coverage despite a significant drop in number. To that aim, Spearman’s correlation coefficients were estimated between *k* and two different statistics, i.e., the mean value of all pairwise molecular distances between the observed alleles (mean PMD), and the mean value of all pairwise peptide-binding distances (mean PPBD) between their corresponding HLA molecules, respectively. We also tested the relationship between these two statistics and both the sample size (*N*) and (to correct for sample size heterogeneity among populations) the allelic richness (ar) using the formula given by El Mousadik and Petit (1996). Finally, the relationship between *k* and the two distance statistics was also explored by simulation. To do so, we generated collections of 46 samples of *N* = 50 individuals (the smallest sample size in the dataset) by bootstrapping from each of the 46 original population samples. Spearman’s correlation coefficients between the simulated *k* and both distances were then estimated for each collection of 46 bootstrapped samples and repeated 2500 times as a tradeoff between computational time and numerical precision to obtain empirical distributions to be compared to the observed correlation coefficients. This approach was expected to correct for sample size disparities and thus indicate whether the sample size affected the observed correlations between *k* and the distances.

Our second approach was to use the HLA class I genotype data of the 6094 individuals to estimate two newly defined parameters, (i) the relative increase of molecular distance (RIMD) and (ii) the relative gain in peptide-binding coverage (RGPBC) for each individual. These two variables estimate the extent of the immune potential of an individual conferred by the two alleles he carries at a given locus. The HLA molecules might exhibit a higher capacity to bind different peptides (i.e., a higher immune potential) because they differ from each other either by a greater molecular distance (allele divergence) or by a greater peptide-binding distance (peptide-binding coverage) when compared to a null situation where these two parameters are equal to zero. For example, a homozygote at locus HLA-A will have a RIMD_A_ of 0, whereas a heterozygote will have a RIMD_A_ varying from 0 (in case of a heterozygote carrying alleles with an identical PBR sequence) to 1 with increasing molecular distances between its two HLA-A alleles. The same rationale applies to estimate RGPBC after replacing the molecular distances by the peptide-binding distances. Interestingly, these two parameters can also be estimated by considering several loci at the same time. For example, a homozygote at both loci HLA-A and HLA-B with same PBR sequence will have a RIMD_AB_ of 0, whereas a heterozygote at any of the two loci, or both, will have a RIMD_AB_ varying from 0 to 1 with increasing mean molecular distances between its four HLA-A and HLA-B alleles and so on for the three loci A, B, and C. Finally, the two parameters can be used at the population, rather than the individual, level: a high mean RIMD between the individuals of a given population indicates that they tend to carry molecularly distant alleles and a high mean RGPBC that they tend to express HLA molecules with elevated peptide-binding distances. Furthermore, if the loci combinations do not have any additional effect, the expectation is that the variance of both RIMD and RGPBC measured on the population samples remains at the same level as for individual loci (or, at least, a reduction is not expected), what provides a statistical test for the null hypothesis of loci acting independently on RIMD and RGPBC. Both parameters thus capture the breadth of functional diversity in populations from an immunological perspective and allow one to compare the obtained profiles to the ones obtained with more conventional measures of diversity such as heterozygosity. Based on these, mean RIMD and RGPBC were estimated in the 46 populations of this study. Linear models followed by Tukey’s HSD post hoc tests were then used to compare these values across the loci, i.e., across the three individual class I loci (A, B, and C), across all pairs of loci (AB, AC, and BC), and across the three loci taken together (ABC). In addition to the “locus” variable, population demography (defined by the two categories RGD and SGD, see above) was also included as an explanatory variable in the models tested (but we did not use the geographic information because of the uneven distribution of samples among the different geographic regions, see Table 1 and Supplementary Material and Methods, Online Resource 1).

Most of the analyses described above were performed with R version 3.1.0 (R Core Team 2014) using the packages coin (Hothorn Torsten 2008), to perform statistical analyses with *p* values provided by Monte Carlo simulations, and ggplot2 (Wickham 2009), for exploratory data analysis and graphics. The various linear models used always accounted for main effects and interactions of the predictor variables.

## Results

### Molecular diversity of the HLA class I genes

#### Distribution of polymorphic residues within the PBR

The results of the independence test between the polymorphic status (M/NS/S) of the codons and their involvement in either pocket-like structures (P/NP) or peptide binding (B/NB) indicate that polymorphic residues are predominantly non-randomly distributed within the PBR of the HLA class I molecules (Table 2). Indeed, except in one case (for HLA-C peptide binding when a correction for multiple tests is applied: *p* = 0.037, n.s. with *α*′ = 0.00625), the independence is significantly rejected (*p* values: 0.0005–0.0045). In all cases, we observe a significant (stdres > |2|) excess of non-synonymous codons (NS) for residues which are either physically located within the pockets of the PBR (P) or outside these pockets but broadly involved in peptide binding (B). By contrast, monomorphic codons (M) are significantly overrepresented for residues which are either located outside the PBR pockets (NP) or not involved in peptide binding (NB). Also, synonymous sites (S, the few ones that were not eliminated when we formatted the data, see “Materials and methods”) are mainly detected at codons coding for residues which are located outside the PBR pockets (NP) or not involved in peptide binding (NB).

**Table 2:** Distribution of polymorphic residues within the PBR

		ABC			HLA-A			HLA-B			HLA-C		
		M	NS	S	M	NS	S	M	NS	S	M	NS	S
Distribution^a^	P	9	25	0	13	20	1	10	24	0	19	15	0
	NP	67	64	18	107	32	10	109	33	7	119	22	8
Stdres^b^	P	−1.97	3.22	−2.13	−3.72	4.36	−0.83	−4.83	5.5	−1.29	−2.93	3.85	−1.38
Chi-square *p* values		0.0045			0.0005			0.0005			0.0005		
Distribution^a^	B	19	43	3	29	33	3	28	35	2	45	19	1
	NB	57	46	15	91	19	8	91	22	5	93	18	7
Stdres^b^	B	−2.51	3.52	−1.76	−4.43	4.98	−0.59	−4.62	4.92	−0.39	−1.44	2.25	−1.39
Chi-square *p* values		0.0025			0.0005			0.0005			0.037		

*M* monomorphic codons, *NS* codons containing at least one non-synonymous polymorphic site, *S* codons containing only synonymous polymorphic site(s), *P* pocket, *NP* non-pocket, *B* binding, *NB* non-binding (see Supplementary material and methods)

^a^Number of codons for each category

^b^Standardized residuals are shown for the P and B categories. The stdres for NP and NB consist in the opposite values to P and B, respectively

#### Amount and distribution of molecular variation within the PBR-coding region

**Fig. 1:**
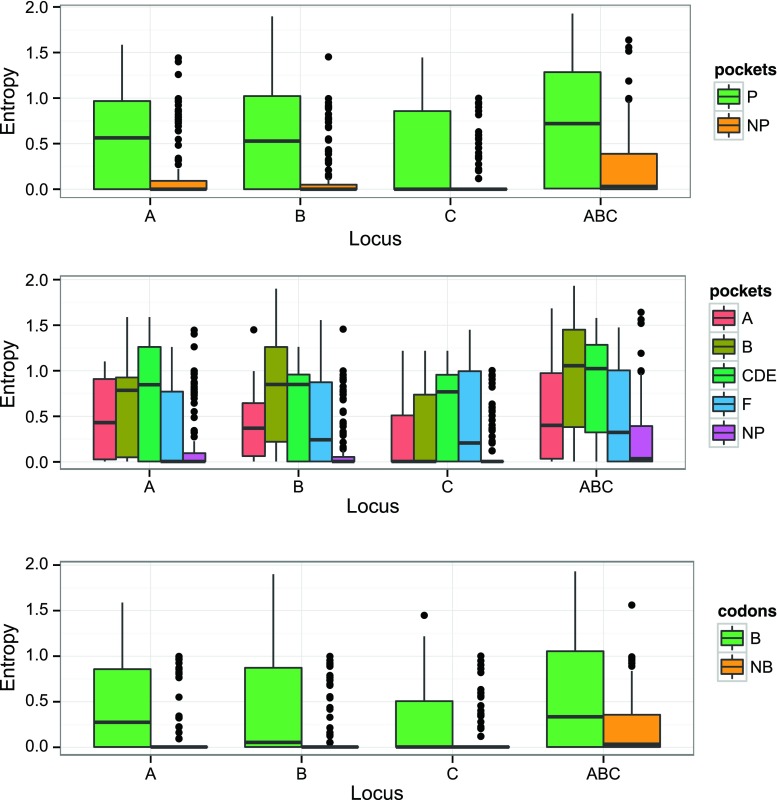
Box and whisker plots of the entropy (*H*_CODON_MAX_) at each of the 183 codons coding for the peptide-binding region of each HLA class I locus HLA-A, HLA-B, and HLA-C; (*top*) when categorized as pocket codons (*P*, *n* = 34) and non-pocket codons (*NP*, *n* = 149); (*middle*) when subdividing the “P” codons into each of the four PBR pockets (*A*, *B*, *C*, *D*, *E*, and *F*); (*bottom*) when categorized as binding codons (*B*, *n* = 65) and non-binding codons (*NB*, *n* = 118). Entropy estimated on the basis of the combined sequence alignments for the three loci is also illustrated (*ABC*). The *boxes* correspond to the interquartile range, the median is the *thick line* inside the box, and whiskers extend up to observations that are outside the box for less than 1.5 times the interquartile range. *Dots* are outliers to these limits

Molecular variation assessed by codon entropy (*H*_CODON_MAX_) within the PBR-coding region (exons 2 and 3) is significantly higher at residues located within (P) than outside (NP) the PBR pockets (Fig. 1 *top* and *p* < 2.2e-16 at A, B, C, and ABC according to Wilcoxon-Mann-Whitney rank sum tests) and at binding (B) than non-binding (NB) residues (Fig. 1 *bottom* and *p* < 2.2e-16 at ABC, A, and B), except at HLA-C (*p* = 0.05). When considering the four pockets A, B, CDE, and F, CDE is the most variable at all loci, and F the less variable except at locus C (Fig. 1 *middle*). Interestingly, codon 116 of locus C exhibits the highest entropy within the F pocket (1.45), followed by codon 80 (0.997), possibly because the corresponding residues assume key functions of HLA-C. Actually, residue 116 is of critical relevance in hematopoietic stem cell transplantation (Ferrara et al. 2001; Pidala et al. 2013), while residue 80 defines the epitopes C1 (with asparagine), recognized by receptors KIR2DL2/3 and KIR2DS4, and C2 (with lysine), recognized by receptors KIR2DL1 and KIR2DS1, respectively (Boyington and Sun 2002; Parham and Moffett 2013).

According to the linear regression model that we used to analyze *H*_CODON_MAX_ in relation to both the “locus” and the “pockets” (i.e., location of residues within or outside the four PBR pockets) variables (Supplementary Table S1a, Online Resource 2), the global *F* statistic is highly significant and both variables are significant predictors of the entropy (*p* < 0.05 or <0.01 for “locusA” to “locusC” and “pocketA” to “pocketF”). Also, the only significant interaction between the two variables involves HLA-C and pocket B (“pocketB:locusC”). According to the Tukey’s HSD post hoc test (Supplementary Table S1b, Online Resource 2), for the “pockets” variable, *H*_CODON_MAX_ is significantly lower at codons defining non-pocket (NP) residues than at those defining any of the four pockets A, B, CDE, and F and significantly higher at codons defining the CDE pocket than at those defining the A pocket (*p* adj <0.05 for “NP-A” to “NP-F” as well as ”CDE-A”); for the “locus” variable, *H*_CODON_MAX_ is significantly higher at ABC than at any of the three individual loci (*p* adj <0.05 for “A-ABC” to “C-ABC”), whereas loci A, B, and C are not significantly different from each other (*p* adj >0.05 for “B-A,” “C-A,” “C-B”). These results are in agreement with the graphs shown in Fig. 1.

In summary, although some differences are revealed for HLA-C, the results confirm that the polymorphic residues are not randomly distributed within the PBR and follow a pattern related to the functional properties of the HLA molecules. Also, in agreement with Hedrick et al. (1991), but using a much larger number of sequences, the variation is significantly higher at the residues involved in peptide binding compared to the other residues of the PBR.

### Functional diversity of the HLA class I genes

#### Patterns of molecular and predicted peptide-binding distances between alleles and molecules

The PMD among the 328 observed HLA class I alleles are shown with density curves in Supplementary Fig. S1a (Online Resource 3). HLA-A and B alleles differ two by two by a large number of nucleotide differences (approximately 40 to 60). The same is true for HLA-A and C alleles, whereas HLA-B and C alleles are more closely related (20 to 50 nucleotide differences). Within each locus, HLA-B exhibits the largest number of nucleotide differences between alleles (up to 47, with a mean of 22.4 and standard deviation of 8.2), followed by HLA-A (up to 39, with a mean of 21.8 ± 8.4). HLA-C alleles are less diverse, with only up to 25 nucleotide differences among each other (mean of 13.6 ± 4.8). These patterns are consistent with HLA class I genes evolution in primates (see “Discussion”).

The density curves of the PPBD predicted with MHCcluster 2.0 between the corresponding 328 molecules are shown in Supplementary Fig. S1b (Online Resource 3 ). The distances between molecules taken from different class I loci are usually (very) high (almost always >0.4). This is also true, but to a lesser extent, between molecules taken within either HLA-A or HLA-B, with skewed density distributions towards high peptide-binding distances at both loci. By contrast, HLA-C molecules exhibit lower PPBD as well as a clear bimodal density distribution, suggesting the existence of two broad groups of molecules differing by their peptide-binding properties.

#### Effect of the number of alleles on molecular distances and peptide-binding coverage

To test the relationship between the number of alleles observed in populations (*k*) and the depth of their immune repertoire, we first checked that *k* was smaller in small-sized and isolated (RGD) than in large and outbred (SGD) populations. The differences between the two groups are significant at each locus taken individually, as well as when we consider the different pairs of loci or even the three loci taken together (*p* values = 2.2e-16 at A, B, C, AB, AC, BC, and ABC according to Wilcoxon-Mann-Whitney rank sum tests and corresponding graphs in Supplementary Fig. S2, Online Resource 3). The correlations between *k* and the mean PMD and PPBD at each locus and for the different locus combinations are given in Table 3. Only in two cases, a highly significant negative correlation is observed (“*r* with *k*” = −0.76, *p* = 7.6e-10 at HLA-C for PMD; “*r* with *k*” = −0.59, *p* = 1.7e-05 at HLA-A for PPBD), suggesting an overall increase in peptide-binding coverage with a decreasing number of alleles. Otherwise, we observe either a significant positive correlation ranging between 0.42 and 0.74 (*p* < 0.01) or no significant correlation (*p* > 0.05). These unexpected results probably arose because the number of observed alleles (*k*) is not independent from the sample size (*N*). We indeed confirmed that *k* and *N* are highly (positively) correlated at all loci (“*r* between *k* and *N*” ranging from 0.53 to 0.65, with all *p* < 0.001, Table 3). This is also shown in Supplementary Fig. S2 (Online Resource 3), more particularly in the case of large outbred (SGD) populations which exhibit more heterogeneous sample sizes (see also Table 1). Therefore, in an attempt to correct for sample size heterogeneity, we tested the correlation between both the mean PMD and the mean PPBD and the allelic richness instead of *k*. However, the results are very similar to those obtained when using *k* (Table 3), suggesting that allelic richness does not correct adequately or sufficiently for sample size heterogeneity. This is why an additional approach using a re-sampling procedure (see “Material and methods”) was applied. The results show that the correlation coefficients observed at A, C, AB, and AC, when the mean PMD is used (Fig. S3a, Online Resource 3), and at A, B, C, and AB, when the mean PPDB is used (Fig. S3b, Online Resource 3), deviate substantially from the empirical distributions obtained through 2500 random samplings, indicating a significant effect of sample size on the allele repertoires of the studied populations. As a consequence, we cannot draw any conclusion on the effect of *k* and ar on peptide-binding coverage.

**Table 3:** Analysis of the allelic repertories

			Mean pairwise peptide-binding distances (PPBD)						Mean pairwise molecular distances (PMD)					
Loci	*r* between *k* and *N*	*p* value	*r* with *N*	*p* value	*r* with *k*	*p* value	*r* with ar	*p* value	*r* with *N*	*p* value	*r* with *k*	*p* value	*r* with ar	*p* value
ABC	0.65	1.1E-06	0.42	3.9E-03	0.43	2.8E-03	–	–	0.45	1.7E-03	0.63	2.8E-06	–	–
AB	0.66	7.3E-07	0.11	0.45	0.02	0.89	–	–	0.34	0.02	0.43	2.5E-03	–	–
AC	0.6	1.2E-05	0.49	4.8E-04	0.39	0.01	–	–	0.51	2.5E-04	0.52	1.9E-04	–	–
BC	0.63	2.3E-06	0.45	1.6E-03	0.42	3.9E-03	–	–	0.29	0.05	0.56	5.6E-05	–	–
A	0.64	1.8E-06	−0.47	9.4E-04	−0.59	1.7E-05	−0.55	7.6E-05	0.43	3.1E-03	0.74	5.4E-09	0.72	1.5E-08
B	0.67	3.6E-07	0.29	0.05	0.14	0.34	0.1	0.51	0.4	0.01	0.55	6.7E-05	0.53	0
C	0.53	1.4E-04	0.05	0.72	−0.22	0.14	−0.2	0.19	−0.46	1.3E-03	−0.76	7.6E-10	−0.71	2.5E-08

*r* correlation coefficient, *N* sample size, *k* number of alleles, *ar* allelic richness (note that it was only possible to estimate this parameter at individual loci and not when using multi-loci data)

#### Effect of the genotypes on molecular distances and peptide-binding coverage

**Fig. 2:**
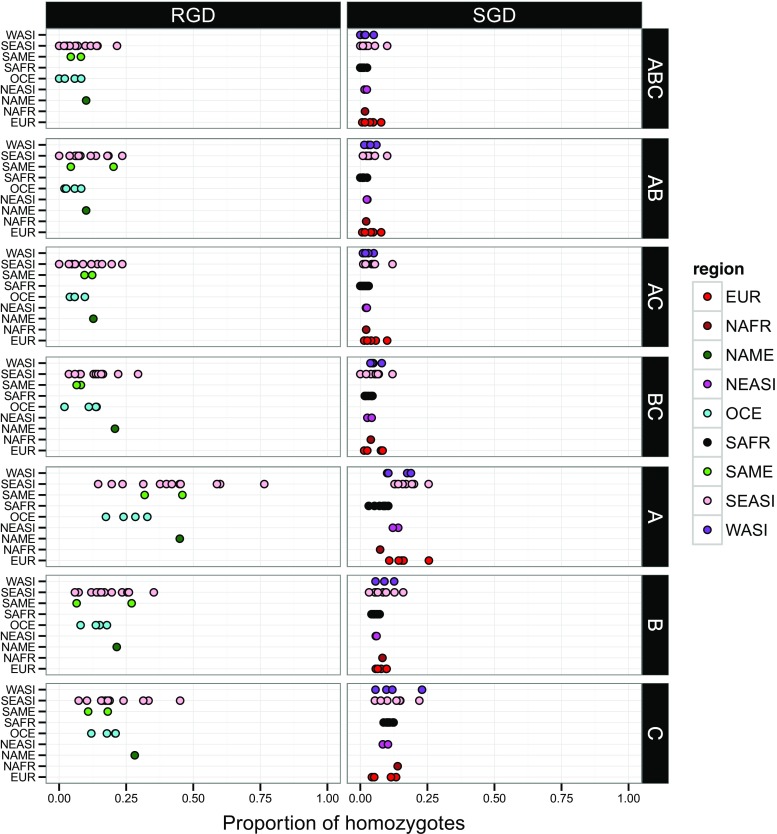
Proportion of homozygotes in 46 human populations at single and multiple loci. The geographic provenance of each population is indicated by a *colored dot*. Populations are subdivided into rapid genetic drift (*RGD*, on the *left plots*) and slow genetic drift (*SGD*, on the *right plots*)

Our last approach investigated the relationship between the polymorphism of the three HLA class I genes and the functionality of the corresponding HLA molecules by using the genotypic rather than the allelic data of the different populations. First, we estimated the proportion of homozygotes at single and multiple loci (e.g., at ABC, individuals were considered as homozygous when they carried only one HLA-A, one HLA-B, and one HLA-C allele) in both small-sized isolated (RGD) and large outbred (SGD) populations. As shown in Fig. 2, higher proportions of homozygotes are found at single loci, especially HLA-A, compared to multiple loci: A (mean of 23.5 %) ≫ C (14.8 %) ≫ B (11.5 %) ≫ BC (7.78 %) ≫ AC (6.17 %) > AB (5.5 %) ≫ ABC (4.33 %) (*p* varying from 6e-13 to 0.005 according to pairwise Wilcoxon rank sum tests after Holm’s adjustment correction for multiple tests, except for AB and AC: corrected *p* value = 0.074). A substantial variation is also observed between populations, both among and within geographic regions (not tested statistically because of disparities in the number of samples tested in each region). Actually, the most striking difference appears between RGD and SGD populations, with a significantly higher homozygosity in the former (*p* = 2.2e-16 at A, B, C, AB, AC, and BC and 3e-04 at ABC, respectively, according to Wilcoxon-Mann-Whitney rank sum tests).

**Fig. 3:**
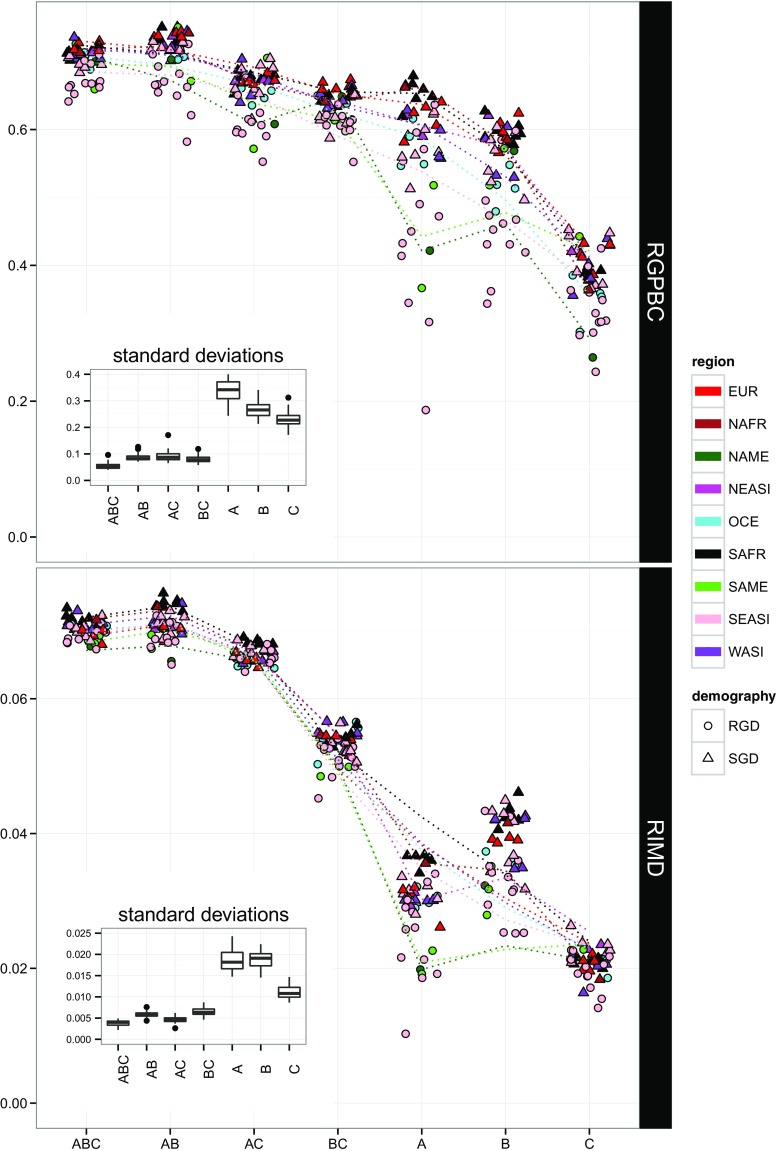
Mean relative gain in peptide binding coverage (*RGPBC*) and mean relative increase in molecular distance (*RIMD*) in 46 human populations. Broad geographic regions are indicated by different colors, while demography is indicated by the shape of the dots (a *circle* for populations characterized by rapid genetic drift (*RGD*) and a *triangle* for populations with slow genetic drift (*SGD*)). Different scales are used on the *y*-axis for both measures. Standard deviations of *RGPBC* and *RIMD* in the populations are provided as insets and represented with boxplots

The mean relative increase in molecular distances (mean RIMD) and the mean relative gain in peptide-binding coverage (mean RGPBC) are shown in Fig. 3 for the 46 populations labelled with their corresponding geographic (region) and demographic (RGD or SGD) information (an alternative representation with density curves is provided in Fig. S4a, b, Online Resource 3). Both RIMD and RGPBC show a steady increase from single locus to multi-locus genotypes (i.e., when moving along *x*-axis from right to left in Fig. 3). The lowest values are observed at HLA-C (except for one outlier population at HLA-A), and maximum values are reached at AB and ABC loci groups (the slight decrease between AB and ABC is not significant, see hereafter). This “functional plateau” is reached by every population, despite the fact that all populations differ from each other by their sets of alleles and frequency distributions (Buhler and Sanchez-Mazas 2011; Mack and Erlich 2006; Riccio et al. 2013; Sanchez-Mazas et al. 2011). The mean RIMD and RGPBC were also analyzed by using a linear regression model considering both “locus” and “demography” as explanatory variables (Table 4). The best model retained for each index is highly significant (large *F* statistics with *p* < 0.01). The results reveal that RIMD and RGPBC differ significantly among loci and groups of loci (*p* < 0.01 for “locusAC” to “locusC” in Table 4 and *p* adj <0.05 in Table S2a and b, Online Resource 2), except RIMD between AB and ABC and RGPBC between A and B, BC and AC, and AB and ABC (*p* adj >0.05 in Tables S2a and b). These observations suggest that the populations reach both a maximum molecular variation (assessed by RIMD) and a maximum peptide-binding coverage (assessed by RGPBC) when the two loci A and B are considered together, whereas locus C has no supplementary effect on these variables when added to A and B (i.e., “locusAB” is not different from the baseline represented by “locusABC,” Table 4). Significant differences of both RIMD and RGPBC are also observed at HLA-A and HLA-B between the two groups of populations defined on a demographic criterion, i.e., RGD and SGD, and (to a lesser degree) of RGPBC at HLA-C (see “locusA:demographySGD,” “locusB:demographySGD,” and “locusC:demographySGD” in Table 2 and the adjusted *p* values in Tables S2a and S2b, Online Resource 2). By contrast, the differences between RGD and SGD populations vanish when multi-locus data are considered: they are only significant (and to a much lesser extent than at single loci) at AB and AC for RGPBC but not in all other cases (i.e., at AB and AC for RIMD and at BC and ABC for both RGPBC and RIMD, see the adjusted *p* values in Tables S2a and S2b, Online Resource 2). A confirmatory pattern is observed when plotting the standard deviations of RGPBC and RIMD (Fig. 3); we obtain very narrow boxplots with low medians at multi-loci (which confirms the reduction of variance expected if loci combinations have an additional effect) in contrast to extended boxplots with much higher medians at each single locus and especially so at HLA-A and B. In other words, these results suggest that, when several HLA loci are considered together, the two groups of populations exhibit equivalent molecular variation and peptide-binding coverage, whereas it is not the case when each locus is considered separately due to significant differences in their homozygosity.

**Table 4:** General linear regression models for mean relative gain in peptide binding coverage (RGPBC) and mean relative increase in molecular distance (RIMD)

	Dependent variable	
	RGPBC	RIMD
locusAB	−0.001	−0.0001
	(0.014)	(0.001)
locusAC	−0.057**	−0.004**
	(0.014)	(0.001)
locusBC	−0.066**	−0.018**
	(0.014)	(0.001)
locusA	−0.205**	−0.044**
	(0.014)	(0.001)
locusB	−0.196**	−0.036**
	(0.014)	(0.001)
locusC	−0.341**	−0.049**
	(0.014)	(0.001)
demographySGD	0.029*	0.001
	(0.013)	(0.001)
locusAB:demographySGD	0.02	0.001
	(0.018)	(0.001)
locusAC:demographySGD	0.018	−0.0003
	(0.018)	(0.001)
locusBC:demographySGD	−0.004	0.001
	(0.018)	(0.001)
locusA:demographySGD	0.106**	0.006**
	(0.018)	(0.001)
locusB:demographySGD	0.067**	0.006**
	(0.018)	(0.001)
locusC:demographySGD	0.032*	0.000
	(0.018)	(0.001)
Constant	0.682**	0.069**
	(0.010)	(0.001)
Observations	322	322
*R* ^2^	0.88	0.98
Adjusted *R* ^2^	0.87	0.98
Residual std. error (*df* = 308)	0.04	0.003
*F* statistic (*df* = 13; 308)	167.724**	1165.377**

Standard errors are provided within parentheses. Baseline groups are “ABC” (for the “locus” explanatory variable) and “rapid genetic drift” (“RGD” for the “demography” explanatory variable)

*SGD* slow genetic drift

*p < 0.05; **p < 0.01

## Discussion

### The genetic variability of HLA class I genes from a functional perspective

Because a high MHC genetic diversity is considered to be critical for adaptive immune processes (Sommer 2005), the significant drop of diversity documented in several small-sized and isolated populations (Buhler and Sanchez-Mazas 2011; Cadavid and Watkins 1997; Chu et al. 2001; Edinur et al. 2013), also revealed in the current population dataset, is intriguing. Such a reduction of genetic variation can be explained by several demographic mechanisms such as genetic drift, bottleneck, or population substructure due to long-term geographic or cultural isolation but also by deterministic forces such as purifying selection systematically eliminating deleterious variants or positive directional selection spreading some advantageous alleles towards fixation (Cavalli-Sforza and Bodmer 1971; Crow and Kimura 1970; Kimura and Crow 1964; Malécot 1975; Wright 1951). In the case of HLA, although balancing selection has usually been invoked to interpret the patterns of molecular variation observed in humans (Buhler and Sanchez-Mazas 2011; Meyer et al. 2006), directional selection affecting HLA frequencies, e.g., during an epidemic, is a likely mechanism (Parham 2009; Wroblewski et al. 2015) provided that many alleles are presumed to confer susceptibility or resistance to infectious diseases (Garamszegi 2014; Hill 1998; Trowsdale 2011). One example is HLA-B*53:01, a protective allele against severe malaria (Hill et al. 1992), that exhibits a marked increase of frequencies in areas of Sub-Saharan Africa where *Plasmodium falciparum* is prevalent (Garamszegi 2014; Testi et al. 2015). However, even in areas where malaria is the main endemic disease, many other pathogens may also be present and a low level of HLA allele diversity may still be very detrimental. In this context, we thus planned to investigate the relationship between the molecular and functional diversity (i.e., the putative ability to present a broad spectrum of peptides) of the 3 HLA class I genes A, B, and C in a large set of 46 populations from different geographic locations and showing contrasted amounts of HLA polymorphism.

We first assessed the relevance of using the huge levels of non-synonymous molecular variation observed in exons 2 and 3 as a sound, although indirect, estimate of the properties of HLA class I molecules at binding antigenic peptides. Indeed, these two exons are the backbone where most of the functional polymorphism has been accumulating, being generated by de novo mutational events or shuffled by recombination and gene conversion events (Martinsohn et al. 1999). By analyzing the codons’ molecular entropy at the three class I genes, we confirmed and generalized the previous suggestion of Hedrick et al. (1991) that non-synonymous sites in exons 2 and 3 are not randomly distributed within the PBR (despite some peculiarities observed at the HLA-C locus) but follow a pattern that is significantly related to functionality. Indeed, these sites are concentrated not only in the codons that define the pocket-like structures of the PBR accommodating the antigenic peptides but also in codons that are defined as critically involved in peptide binding (Bjorkman et al. 1987; Chelvanayagam 1996; Kangueane et al. 2001; Reche and Reinherz 2003), despite their physical location outside of these pockets. This pattern is concordant with the hypothesis that balancing selection is a main force maintaining high levels of molecular variation in the PBR (Bitarello et al. 2015; Hedrick et al. 1991; Hughes and Nei 1988). As a complementary and original approach, we used peptide-binding predictions which allowed us to show that the patterns of pairwise molecular distances among alleles are similar to the patterns of predicted peptide-binding distances among their corresponding molecules. The latter were assessed by NetMHCpan (Hoof et al. 2008), one of the best performing algorithms currently available for class I binding predictions (Trolle et al. 2015; Zhang et al. 2009, 2012), and which was recently integrated into a method, MHCcluster (Thomsen et al. 2013), allowing to perform functional clustering of HLA class I molecules. In accordance with our results, a recent study indicates that the amino acid positions that mostly alter peptide binding are the highly polymorphic ones, while other codons have much less or no influence at all on the peptide repertoire (van Deutekom and Keşmir 2015). In light of these observations, both types of distances (molecular and peptide binding) were retained in this study as independent and complementary measures of the HLA class I functional diversity, i.e., the ability to present a broad spectrum of antigenic peptides.

### Explaining the evolution of HLA class I polymorphism by a model of joint divergent asymmetric selection

Our objective was then to compare this functional diversity among the 46 populations of our study. To that aim, we first defined two original indices, the RIMD and the RGPBC, allowing to compare the levels of functional variation among individuals, conditional of their heterozygous state (accounting for allele divergence or DAA) and of the alleles carried at one, two, or the three classical HLA class I loci (A, B, C) considered together. While the mechanisms of natural selection occur at the level of the individuals, signals of the process of natural selection are only visible at the level of the populations. We thus expanded the functional comparisons by computing mean values of RIMD and RGPBC in each population, again by considering successively each locus separately, the different pairs of loci and the three loci together. The most outstanding result was that while small-sized and isolated (RGD) populations differ in their functional diversity (in terms of both molecular variation and peptide-binding coverage) from large outbred (SGD) populations at individual class I genes (and particularly so at HLA-A and HLA-B), all populations share similar levels of functional diversity when the three loci (ABC) are considered together. In other words, a similar amount of functionally relevant HLA diversity appears to be maintained at the three HLA class I genes taken together in every population, irrespective of a drop of diversity at single loci due to demographic events or directional selection. Based on this essential result, we thus propose a model of joint divergent asymmetric selection acting on (the three classical genes of) HLA class I as a whole.

A form of DAA selection thus appears to favor combinations of functionally divergent alleles at multiple loci in different populations, probably as a general mechanism to hamper pathogens evading immune recognition (Lenz 2011; Potts and Slev 1995). Moreover, the diversifying effect of selection is likely to reach a maximal threshold, here evidenced by the “functional plateaus” shown in Fig. 3. This latest observation parallels a suggestion by Lau et al. of an upper limit to sequence divergence generated by the DAA model at HLA class II locus, HLA-DRB1 (Lau et al. 2015). One reason invoked for explaining this limit is that a too high sequence divergence between HLA molecules would reduce the T cell repertoire during thymus maturation (Lau et al. 2015; Lenz 2011), in the same way to what has been proposed for a too high number of MHC molecules (Nowak et al. 1992; Woelfing et al. 2009). Some empirical evidence for this hypothesis comes from studies of mate choice in fish, where intermediate rather than maximum MHC sequence dissimilarity would be preferred (Forsberg et al. 2007; Lenz et al. 2009; Nowak et al. 1992; Woelfing et al. 2009). This hypothesis is also retained by Chappell et al. (2015) to explain why promiscuous (i.e., generalist) MHC molecules, presenting a great variety of antigenic peptides, exhibit significantly lower levels of expression than fastidious (i.e., specialist) ones, thus allowing survival of enough T cell clones during negative selection in the thymus. In addition to the model of joint divergent asymmetric selection, the present study thus proposes for the first time an upper limit to both sequence divergence—and hence peptide-binding distances—for HLA class I alleles.

In line with the above mentioned observations, a substantial overlap between the peptides bound by different HLA molecules at one or several loci is often observed, even when these molecules exhibit a large variability in the number of peptides that they are able to bind (Lenz 2011; Rao et al. 2011). This binding overlap, or promiscuity (in the sense of non-specific binding), may lead to a much reduced HLA functional polymorphism than anticipated (Rao et al. 2011) and may explain the currently observed “plateaus.” Actually, some level of redundancy is essential to prevent pathogens evading immune recognition (Potts and Slev 1995). Interestingly, it has been recently proposed that MHC class I molecules can either have promiscuous or fastidious binding properties (i.e., subdividing into generalist and specialist molecules, respectively) as alternative strategies for resistance against different pathogenic strains (Chappell et al. 2015). Furthermore, contrasting levels of peptide-binding promiscuity have also been invoked to explain the distinct patterns of genetic variation observed in relation to pathogen richness at HLA-DQA1 and DQB1, on the one hand, and at HLA-A, B, C, and DRB1, on the other hand (Sanchez-Mazas et al. 2012).

One question that remains open is the timescale of the joint divergent asymmetric selection acting on the HLA loci, as suggested in this study. As the RGPBC and RIMD variables pertain to a genotype-level aspect of variation, it is probable that only selection operating in relatively recent timescales can be detected. However, part of our approach incorporates a molecular level of variation into the equation and might thus allow inferring balancing selection at a longer term, a bit similar to what is proposed for the Tajima’s *D* statistic (Garrigan and Hedrick 2003). In any case, measuring the timescale of balancing selection in populations is a complex issue and warrants further and more detailed analyses.

### The peculiar evolution of HLA-C

Although focusing only on class I genes, the present investigation also uncovers some fundamental similarities or dissimilarities among different HLA loci. Indeed, the pair AB exhibits the greatest increase of functional variation, suggesting that a joint role of these two genes is a critical characteristic of class I peptide presentation. This is concordant with the finding of a general complementarity of binding motifs between HLA-A and B molecules (Rao et al. 2013). By contrast, the implication of HLA-C in this process is less obvious, as the inclusion of this locus does not add anything to the maximal increase of functional variation seen at HLA-AB (the mean RIMD and RGPBC reached by the ABC trio are not significantly different from those reached by the AB pair). Moreover, while some differences in RGPBC can be retrieved between RGD and SGD populations (but to a much lesser extent than at HLA-A and B), the RIMD values estimated for HLA-C are much more similar between these two groups than for the other loci. Other peculiar results characterize the HLA-C locus: the molecular entropy estimated in the PBR provides a weaker signal for polymorphic codons than for HLA-A and B; the pocket-like structure exhibits lower levels of variation in pocket B compared to HLA-A and B and probably a more prominent role of pockets CDE and F at defining the peptide-binding properties of HLA-C molecules; and finally, the peptide-binding distances predicted with MHCcluster suggest a subdivision of HLA-C molecules into two broad groups regarding their peptide presentation properties.

Our hypothesis is that HLA-C does not contribute substantially to the diversification of class I peptide presentation. Actually, the HLA-C polymorphism is peculiar in several respects: it exhibits very balanced frequency distributions according to selective neutrality tests and the patterns of molecular diversity observed in human populations differ markedly from those of the other loci (Buhler and Sanchez-Mazas 2011; Qutob et al. 2011; Solberg et al. 2008); the HLA-C gene also distinguishes itself by a differential pattern of expression, both in lower levels on cell surface (McCutcheon et al. 1995; Neisig et al. 1998) and in tissue distribution (Apps et al. 2009; King et al. 1996); and finally, from a functional point of view, HLA-C is the prominent and specialized ligand for killer cell immunoglobulin-like receptors (KIR) expressed on natural killer (NK) cells (Norman et al. 2013; Parham 2005), with C1 and C2 allotypes being recognized by several activator and inhibitory receptors and maintained (probably by balancing selection) in all extant human populations (Parham et al. 2012). HLA-C has thus likely evolved in a very different way than the other class I loci, despite its more recent origin. In primates, MHC-C is thought to have arisen from a duplication of a MHC-B ancestral gene after the divergence of apes and Old World monkeys (Adams and Parham 2001; Fukami-Kobayashi et al. 2005). One stimulating hypothesis to explain the peculiar characteristics of HLA-C molecular diversity observed in the present study is that, following the duplication from a MHC-B ancestor, MHC-C rapidly acquired new functions related to its role as a specialized KIR ligand (see (Parham and Moffett 2013) and references therein) and did not assume an equivalent role as MHC-A and MHC-B in peptide presentation. This study thus also provides the first evidence based on the analysis of HLA sequence diversity that the HLA genomic region underwent a mechanism of birth and death evolution whereby recently duplicated genes acquire new functions, as theoretically proposed for the multigene families of the immune system (Nei and Hughes 1992; Nei and Rooney 2005).

## Conclusion

The HLA polymorphism is characterized by an extraordinary amount of molecular diversity that has since many decades been supposed to play a crucial role in immunity. However, the evolutionary models that have been proposed so far, in particular balancing selection in the form of heterozygous advantage, have not been able to explain some marked differences of diversity between small-sized isolated and large outbred populations, the former being theoretically disadvantaged due to a much lower heterozygosity. Here we reconcile the two kinds of observations by proposing an original model of joint divergent asymmetric selection of the HLA class I genes, which suggests that the lack of diversity at individual loci, as observed in populations submitted to rapid genetic drift or to positive selection due to disease associations, is counter-balanced by complementary peptide-binding properties (due to molecularly divergent alleles) of the molecules coded by several loci. Moreover, while this model seems robust for the two genes HLA-A and HLA-B playing together, the diversity of HLA-C does not increase significantly the HLA class I peptide-binding potential, suggesting that this locus assumes a more important role in its KIR-related functions acquired by birth-and-death evolution. The results presented here provide a framework to conciliate disparate observations and improve considerably our comprehension of the adaptive immune response at the population level.

## Electronic supplementary material

Below is the link to the electronic supplementary material.

Online resource 1 Supplementary material and methods (PDF 119 kb)

Online resource 2 Supplementary Tables S1 (**a** and **b**) and S2 (**a** and **b**) (PDF 172 kb)

Online resource 3 Supplementary Figs. S1 (**a** and **b**), S2, S3 (**a** and **b**), and S4 (**a** and **b**) (PDF 182 kb)
