# Supplementary material for: HLA class I molecular variation and peptide-binding properties suggest a model of joint divergent asymmetric selection
Source: Immunogenetics. 2016 May 27;68(6):401–16. doi: 10.1007/s00251-016-0918-x (PMC4911380; doi:10.1007/s00251-016-0918-x)
Supplement: Supplementary file 1 — Supplementary material and methods (PDF 119 kb) [file 251_2016_918_MOESM1_ESM.pdf]

Supplementary material and methods

Population data

Population samples typed simultaneously at the 3 classical class I loci HLA-A, HLA-B and HLA-C were taken from the Gene[VA] database. Most of these data were gathered during past International Histocompatibility and Immunogenetics Workshops (Bodmer et al. 1997; Mack et al. 2006; Nunes et al. 2010; Riccio et al. 2013). The filtering criteria were as follow: (a) a minimal sample size of 50 individuals and (b) molecular characterization at 2nd-field level of resolution with no ambiguities reported. To increase the number of population samples used in the analysis, we also retained four population samples (all of them with n > 100) containing up to 5 individuals with typing ambiguities which were discarded from the analysis. Working with data defined at 2nd-field level (i.e. protein level of resolution) was mandatory to be able to perform peptide-binding predictions with the MHCcluster 2.0 server (Thomsen et al. 2013). A total of 6094 individuals from 46 populations were finally retained after the filtering (see Table 1 in the manuscript and Table SMM1 with detailed population information at the end of this document). The number of populations available for each geographic region (Europe (EUR), North Africa (NAFR), Sub-Saharan Africa (SAFR), West Asia (WASI), North-East Asia (NEASI), North America (NAME), South America (SAME), South-East Asia (SEASI) and Oceania (OCE)) is quite unbalanced with an over-representation of populations from South-Eastern Asia (almost half of the total). Populations were also subdivided according to their assumed demographic history through either rapid genetic drift (RGD), for small-sized and isolated populations, or slow genetic drift (SGD) for the others (i.e. large outbred populations). This allowed us to work with a more balanced set of 19 RGD and 27 SGD populations, respectively (Table 1 and Table SMM1).

Statistical analyses

Characterizing the HLA class I molecular diversity at the peptide-binding regions

Sequence alignments of exons 2 and 3 of the three HLA class I genes A, B and C were downloaded from the IMGT/HLA database (Robinson et al. 2015) and pre-formatted to 2nd-field level of resolution, as described by Buhler and Sanchez-Mazas (Buhler and Sanchez-Mazas 2011); this process eliminated most synonymous polymorphic sites from the sequences, in agreement with what is expected from the allele-name assignment (Marsh et al. 2010). These sites were replaced in the sequences by a "?" for missing data. All molecular analyses were performed on the 328 HLA class I alleles observed in the 46 available population samples (86, 179 and 63 alleles at HLA-A, B and C, respectively). Nucleotide sites with more than 5% missing data among these alleles were discarded from all subsequent analyses, but this represented only 9 out of 166 polymorphic sites in exons 2 and 3, meaning that the relevant information was kept despite the mandatory pre-treatment of the sequences. In addition, the 5% threshold was determined on the basis of the combined HLA-A, B, C sequence alignment and not for individual loci, thus minimizing any possible heterogeneity in the number of sites among the loci regarding missing data. Pairwise molecular distances (PMD) among alleles were estimated by counting the number of nucleotide differences between their corresponding sequences using Arlequin 3.11 (Excoffier and Lischer 2010).

Shannon entropy (Reche and Reinherz 2003; Shannon 1948) was used as a measure of variability at each nucleotide site (H_{DNA} hereafter), both at each locus separately and by considering the combined ABC alignment. H_{DNA} at sites containing less than 5% missing data was calculated on the basis of the observed A, T, G and C proportions (ignoring the presence of missing data at these sites), while H_{DNA} at positions with more than 5% missing data was not taken into account. To relate the molecular diversity (at exons 2 and 3) of HLA alleles to the peptide-binding properties of their corresponding HLA molecules (defined by amino acid

changes in the peptide-binding region, PBR), all 183 codons of these two exons were characterized by several criteria.

- First, the maximal value of entropy at each codon (H_{CODON_MAX} hereafter) was estimated as the maximum value of H_{DNA} at its three nucleotide positions. This provided a conservative estimate of the molecular variation at each codon. The entropy based on the amino acid alignments downloaded from IMGT/HLA database for the same loci was also estimated (H_{AA}) allowing to categorize codons as containing at least one non-synonymous site ($H_{CODON\_MAX}>0$ and $H_{AA}>0$, label NS), only synonymous site(s) ($H_{CODON\_MAX}>0$ and $H_{AA}=0$, label S) or as being monomorphic ($H_{CODON\_MAX}=0$ and $H_{AA}=0$, label M). Due to the pre-formatting of sequences described above, only 11, 7 and 8 codons were labelled as synonymous (S) at HLA-A, B and C, respectively. This category of codons is not relevant for this work because such codons do not code for polymorphic residues in the PBR and also because all residues involved (except residue 24 at HLA-A) are located outside of the PBR pockets. In addition, we ensured that H_{CODON_MAX} was appropriately estimated for NS codons by not considering the H_{DNA} value of any site with a putative synonymous site remaining from the pre-formatting of sequences.
- Second, the codons were classified as coding or not coding for the residues forming the 6 pocket-like structures (A, B, C, D, E and F) of the PBR as defined by the crystallographic study of Saper et al. (Saper et al. 1991). These pockets accommodate the amino acid residues of the antigenic peptides presented by the HLA molecules. Similarly to Francisco et al. (Francisco et al. 2015), the 3 smaller central pockets C, D and E were regrouped into a single “CDE” pocket of comparable size (i.e. according to the number of residues) to A, B and F pockets for the analyses. As a result, 34 codons were labelled P (for pocket) and 149 NP (for non-pocket) (Table SMM2).

- Third, an additional set of 31 codons has been described as being involved in peptide binding by HLA class I molecules on the basis of common or specific biological, chemical and/or physical properties (Bjorkman et al. 1987; Chelvanayagam 1996; Kangueane et al. 2001; Reche and Reinherz 2003). This broader definition of peptide-binding residues was used to classify the codons into two additional categories, binding (B) and non-binding (NB); of the 183 codons, 65 were labelled B and 118 NB (Table SMM2).

Predicting peptide-binding distances between HLA class I alleles

In order to speed up MHCcluster 2.0 calculation and due to run time limitations on the server using such a large number of HLA molecules simultaneously, the number of bootstrap calculations was reduced from the default value of 100 to 10. The number was chosen, after discussion with the author of MHCcluster, in order to obtain valid results without affecting the accuracy of the displayed functional similarities (Morten Nielsen, personal communication) and the results were checked by comparing the binding distances obtained with a subset of 234 HLA-A, B and C molecules, running the program twice, once with 10 and a second time with 100 bootstrap calculations (Mantel test: $r=0.9994$, $p<0.0001$). Furthermore, in order to verify that the 328 class I molecules used in this analysis were not peculiar in terms of their predicted pairwise binding distances (e.g. for some unknown reason, the subset used in this study could be biased towards molecules that require more extrapolation from experimental affinity data than expected), we compared the binding distances observed with those of samples consisting of 86, 179 and 63 molecules randomly selected among the 886, 1412 and 617 HLA-A, B and C molecules defined in MHCcluster 2.0. This approach confirmed that the observed functional distances were very similar to the ones obtained with random sets of HLA class I molecules (results not shown).

Bibliographic references

Bjorkman PJ, Saper MA, Samraoui B, Bennett WS, Strominger JL, Wiley DC (1987) Structure of the human class I histocompatibility antigen, HLA-A2. Nature 329*:*506-512

Bodmer J, Cambon-Thomsen A, Hors J, Piazza A, Sanchez-Mazas A (1997) Anthroplogy report. Introduction. *In* Charron D (ed.) Proc 12th Int Histocompatibility Workshop and Conference. Paris: EDK

Buhler S, Sanchez-Mazas A (2011) HLA DNA Sequence Variation among Human Populations: Molecular Signatures of Demographic and Selective Events. PLoS ONE 6*:*e14643

Chelvanayagam G (1996) A roadmap for HLA-A, HLA-B, and HLA-C peptide binding specificities. Immunogenetics 45*:*15-26

Excoffier L, Lischer HEL (2010) Arlequin suite ver 3.5: a new series of programs to perform population genetics analyses under Linux and Windows. Molecular Ecology Resources 10*:*564-567

Francisco RdS, Buhler S, Nunes JM, Bitarello BD, França GS, Meyer D, Sanchez-Mazas A (2015) HLA supertype variation across populations: new insights into the role of natural selection in the evolution of HLA-A and HLA-B polymorphisms. Immunogenetics 67*:*651-663

Kangueane P, Sakharkar MK, Kolatkar PR, Ren EC (2001) Towards the MHC-Peptide combinatorics. Human Immunology 62*:*539-556

Mack SJ, Tsai Y, Sanchez-Mazas A, Erlich HA (2006) 13th International Histocompatibility Workshop Anthropology / Human Genetic Diversity Joint Report - Chapter 3: Anthropology / human genetic diversity population reports. *In* Hansen JA (ed.) Immunobiology of the Human MHC: Proceedings of the 13th International Histocompatibility Workshop and Conference. IHWG Press, Seattle, WA

Marsh SGE, Albert ED, Bodmer WF, Bontrop RE, Dupont B, Erlich HA, Fernández-Viña M, Geraghty DE, Holdsworth R, Hurley CK, Lau M, Lee KW, Mach B, Maiers M, Mayr WR, Müller CR, Parham P, Petersdorf EW, Sasazuki T, Strominger JL, Svejgaard A, Terasaki PI, Tiercy JM, Trowsdale J (2010) Nomenclature for factors of the HLA system, 2010. Tissue Antigens 75*:*291-455

Nunes JM, Riccio ME, Buhler S, Di D, Currat M, Ries F, Almada AJ, Benhamamouch S, Benitez O, Canossi A, Fadhlaoui-Zid K, Fischer G, Kervaire B, Loiseau P, De Oliveira DCM, Papasteriades C, Piancatelli D, Rahal M, Richard L, Romero M, Rousseau J, Spiroski M, Sulcebe G, Middleton D, Tiercy JM, Sanchez-Mazas A (2010) Analysis of the HLA population data (AHPD) submitted to the 15th International Histocompatibility/Immunogenetics Workshop by using the Gene[rate] computer tools accommodating ambiguous data (AHPD project report). Tissue Antigens 76*:*18-30

Reche PA, Reinherz EL (2003) Sequence Variability Analysis of Human Class I and Class II MHC Molecules: Functional and Structural Correlates of Amino Acid Polymorphisms. Journal of Molecular Biology 331*:*623-641

Riccio ME, Buhler S, Nunes JM, Vangenot C, Cuénod M, Currat M, Di D, Andreani M, Boldyreva M, Chambers G, Chernova M, Chiaroni J, Darke C, Di Cristofaro J, Dubois V, Dunn P, Edinur HA, Elamin N, Eliaou JF, Grubic Z, Jaatinen T, Kanga U, Kervaire B, Kolesar L, Kunachiwa W, Lokki ML, Mehra N, Nicoloso G, Paakkanen R, Voniatis DP, Papasteriades C, Poli F, Richard L, Romón Alonso I, Slavčev A, Sulcebe G, Suslova T, Testi M, Tiercy JM, Varnavidou A, Vidan-Jeras B, Wennerström A, Sanchez-Mazas A (2013) 16th IHIW: Analysis of HLA Population Data, with updated results for 1996 to 2012 workshop data (AHPD project report). International Journal of Immunogenetics 40*:*21-30

Robinson J, Halliwell JA, Hayhurst JD, Flicek P, Parham P, Marsh SGE (2015) The IPD and IMGT/HLA database: allele variant databases. Nucleic Acids Research 43*:*D423-D431

Saper MA, Bjorkman PJ, Wiley DC (1991) Refined structure of the human histocompatibility antigen HLA-A2 at 2.6 Å resolution. Journal of Molecular Biology 219*:*277-319
Shannon C (1948) A mathematical theory of communication. Bell System Tech. J. 27*:*379-423
Thomsen M, Lundegaard C, Buus S, Lund O, Nielsen M (2013) MHCcluster, a method for functional clustering of MHC molecules. Immunogenetics 65*:*655-665

Table SMM1 Detailed population information

Source_Region_Population	N	Demography
13WS-32_EUR_Czechs	104	SGD
13WS-33_EUR_Finns	90	SGD
13WS-35_EUR_Irish	999	SGD
15WS-12_EUR_Greeks	139	SGD
16WS-44_EUR_Greeks	231	SGD
16WS-35_NAFR_Sudanese	230	SGD
13WS-143_NAME_Yupik	149	RGD
13WS-118_NEASI_Koreans	191	SGD
13WS-119_NEASI_Tuva	165	SGD
13WS-111_OCE_Samoans	50	RGD
13WS-113_OCE_Australian-aborigines-Cape-York	86	RGD
13WS-114_OCE_Australian-aborigines-Groote-Eylandt	73	RGD
13WS-116_OCE_Australian-aborigines-Yuendumu	190	RGD
13WS-1_SAFR_Dogons	129	SGD
13WS-10_SAFR_Zulu	96	SGD
13WS-2_SAFR_Kenyans-Luo	265	SGD
13WS-3_SAFR_Kenyans-Nandi	240	SGD
13WS-4_SAFR_Kenyans	112	SGD
13WS-7_SAFR_Shona	222	SGD
13WS-8_SAFR_Ugandans	161	SGD
13WS-146_SAME_Bari	74	RGD
13WS-148_SAME_Guarani-Kaiowa	138	RGD
13WS-104_SEASI_Filipinos	94	SGD
13WS-106_SEASI_Ivatan	50	SGD
13WS-72_SEASI_Ami	98	RGD
13WS-73_SEASI_Atayal	106	RGD
13WS-74_SEASI_Bunun	101	RGD
13WS-75_SEASI_Chinese	279	SGD
13WS-76_SEASI_Hakka	55	SGD
13WS-80_SEASI_Malay	92	SGD
13WS-81_SEASI_Minnan	102	SGD
13WS-83_SEASI_Okinawans	104	SGD
13WS-84_SEASI_Paiwan	51	RGD
13WS-85_SEASI_Pazeh	55	RGD
13WS-86_SEASI_Puyuma	50	RGD
13WS-87_SEASI_Rukai	50	RGD
13WS-89_SEASI_Saisiyat	51	RGD
13WS-91_SEASI_Siraya	51	RGD
13WS-92_SEASI_Thais	92	SGD
13WS-94_SEASI_Toroko	55	RGD
13WS-95_SEASI_Tsou	51	RGD
13WS-96_SEASI_Yami-Tao	50	RGD
13WS-34_WASI_Georgians	103	SGD
13WS-58_WASI_Druzes	100	SGD
13WS-59_WASI_Israeli-Jews	67	SGD
13WS-61_WASI_Indians-New-Dehli	53	SGD

N: sample size; SGD : slow genetic drift; RGD: rapid genetic drift.

Sources: 13th, 15th and 16th International Histocompatibility and Immunogenetics Workshops.

EUR: Europe, NAFR: Northern Africa, NAME: Northern America, NEASI: North-eastern Asia, OCE: Oceania, SAFR: Sub-Saharan Africa, SAME: Southern America, SEASI: South-eastern Asia, WASI: Western Asia.

Table SMM2 Codons coding for residues located in the PBR pockets and involved in peptide binding

	N	Codons
Binding*	65	5, 7, 9, 11, 22, 24, 25, 26, 33, 34, 35, 36, 45, 58, 59, 62, 63, 65, 66, 67, 69, 70, 72, 73, 74, 76, 77, 79, 80, 81, 83, 84, 95, 96, 97, 99, 100, 114, 116, 117, 118, 123, 124, 133, 142, 143, 146, 147, 149, 150, 151, 152, 154, 155, 156, 158, 159, 160, 162, 163, 164, 166, 167, 170, 171
Pocket A	10	5, 7, 59, 63, 66, 99, 159, 163, 167, 171
Pocket B	11	7, 9, 24, 25, 34, 45, 63, 66, 67, 70, 99
Pockets CDE	13	9, 70, 73, 74, 97, 99, 114, 147, 152, 155, 156, 159, 160
Pocket F	9	77, 80, 81, 84, 116, 123, 143, 146, 147

N: number of codons on a total of 183 PBR codons.

*: codons that have been described as being involved in the peptide binding properties of HLA class I molecules on the basis of common or specific biological, chemical and/or physical properties (Bjorkman et al. (1987), Chelvanayagam (1996), Kangueane et al. (2001), Reche and Reinherz (2003)).
