# Supplementary material for: HLA class I molecular variation and peptide-binding properties suggest a model of joint divergent asymmetric selection
Source: Immunogenetics. 2016 May 27;68(6):401–16. doi: 10.1007/s00251-016-0918-x (PMC4911380; doi:10.1007/s00251-016-0918-x)
Supplement: Supplementary file 2 — Supplementary Tables S1 (a and b) and S2 (a and b) (PDF 172 kb) [file 251_2016_918_MOESM2_ESM.pdf]

Table S1a General linear regression model for the molecular entropy (H_{CODON_MAX}).

	Dependent variable
	entropy
locusA	-0.092*
	(0.044)
locusB	-0.130**
	(0.044)
locusC	-0.161**
	(0.044)
pocketA	0.338**
	(0.123)
pocketB	0.710**
	(0.118)
pocketCDE	0.544**
	(0.109)
pocketF	0.308*
	(0.129)
pocketA:locusA	-0.03
	(0.174)
pocketB:locusA	-0.28
	(0.167)
pocketCDE:locusA	0.03
	(0.154)
pocketF:locusA	-0.05
	(0.183)
pocketA:locusB	-0.03
	(0.174)
pocketB:locusB	-0.07
	(0.167)
pocketCDE:locusB	-0.09
	(0.154)
pocketF:locusB	0.11
	(0.183)
pocketA:locusC	-0.13
	(0.174)
pocketB:locusC	-0.485**
	(0.167)
pocketCDE:locusC	-0.06
	(0.154)
pocketF:locusC	0.08
	(0.183)
Constant	0.257**
	(0.031)
Observations	768
R^2	0.23
Adjusted R^2	0.21
Residual Std. Error	0.377 (df=748)
F Statistic	11.553** (df=19; 748)

Note (1): *p<0.05;**p<0.01

Note (2): standard errors are provided within parentheses

Note (3): baseline groups are 'ABC' (for the 'locus' explanatory variable) and 'NP' (non-pocket for the 'pocket' explanatory variable)

Table S1b Tukey's HSD post-hoc test.

		Difference of means	lwr	upr	p adj
locus	A-ABC	-0.11	-0.21	-0.01	0.02
	B-ABC	-0.14	-0.24	-0.04	0.0024
	C-ABC	-0.2	-0.3	-0.1	0.000003
	B-A	-0.03	-0.13	0.07	0.9
	C-A	-0.09	-0.19	0.01	0.11
	C-B	-0.06	-0.16	0.04	0.4
pockets	B-A	0.21	-0.01	0.44	0.07
	CDE-A	0.22	0.01	0.44	0.04
	F-A	0.05	-0.18	0.29	0.97
	NP-A	-0.29	-0.46	-0.12	0.00003
	CDE-B	0.01	-0.2	0.22	1
	F-B	-0.16	-0.39	0.07	0.32
	NP-B	-0.5	-0.66	-0.34	0
	F-CDE	-0.17	-0.39	0.05	0.22
	NP-CDE	-0.51	-0.66	-0.36	0
	NP-F	-0.34	-0.52	-0.16	0.000002

lwr: lower end point of the interval, upr: upper end point, p adj: p-value after adjustment for the multiple comparisons.

Table S2a Tukey's HSD post-hoc test for the relative gain in peptide binding coverage (RGPBC).

		Difference of means	lwr	upr	p adj
locus	AB-ABC	0.011	-0.016	0.038	0.89
	AC-ABC	-0.047	-0.074	-0.020	8.41E-06
	BC-ABC	-0.068	-0.095	-0.041	1.84E-11
	A-ABC	-0.143	-0.169	-0.116	1.23E-12
	B-ABC	-0.157	-0.184	-0.130	1.23E-12
	C-ABC	-0.322	-0.349	-0.295	1.23E-12
	AC-AB	-0.058	-0.085	-0.031	1.28E-08
	BC-AB	-0.079	-0.106	-0.052	1.30E-12
	A-AB	-0.154	-0.180	-0.127	1.23E-12
	B-AB	-0.168	-0.195	-0.141	1.23E-12
	C-AB	-0.333	-0.360	-0.306	1.23E-12
	BC-AC	-0.021	-0.048	0.006	0.25
	A-AC	-0.096	-0.123	-0.069	1.28E-12
	B-AC	-0.110	-0.137	-0.083	1.23E-12
	C-AC	-0.275	-0.302	-0.248	1.23E-12
	A-BC	-0.075	-0.102	-0.048	1.38E-12
	B-BC	-0.089	-0.116	-0.062	1.29E-12
	C-BC	-0.254	-0.281	-0.228	1.23E-12
	B-A	-0.014	-0.041	0.013	0.71
	C-A	-0.180	-0.206	-0.153	1.23E-12
	C-B	-0.165	-0.192	-0.138	1.23E-12
demography	SGD-RGD	0.063	0.053	0.072	1.23E-12
locus:demography§	ABC:SGD-ABC:RGD	0.029	-0.015	0.073	0.63
	AB:SGD-AB:RGD	0.048	0.004	0.092	0.02
	AC:SGD-AC:RGD	0.047	0.003	0.091	0.03
	BC:SGD-BC:RGD	0.025	-0.019	0.069	0.81
	A:SGD-A:RGD	0.135	0.091	0.179	1.36E-12
	B:SGD-B:RGD	0.096	0.052	0.140	1.58E-10
	C:SGD-C:RGD	0.060	0.016	0.104	0.0004

lwr: lower end point of the interval, upr: upper end point, p adj: p-value after adjustment for the multiple comparisons, RGD: rapid genetic drift, SGD: slow genetic drift.

§: only the RGD-SGD comparisons within each locus are shown.

Table S2b Tukey's HSD post-hoc test for the relative increase in molecular distance (RIMD).

		Difference of means	lwr	upr	p adj
locus	AB-ABC	0.001	-0.001	0.002	0.95
	AC-ABC	-0.004	-0.006	-0.002	7.90E-09
	BC-ABC	-0.017	-0.019	-0.016	1.23E-12
	A-ABC	-0.041	-0.043	-0.039	1.23E-12
	B-ABC	-0.033	-0.035	-0.031	1.23E-12
	C-ABC	-0.049	-0.051	-0.048	1.23E-12
	AC-AB	-0.004	-0.006	-0.003	1.53E-11
	BC-AB	-0.018	-0.020	-0.016	1.23E-12
	A-AB	-0.042	-0.043	-0.040	1.23E-12
	B-AB	-0.033	-0.035	-0.032	1.23E-12
	C-AB	-0.050	-0.052	-0.048	1.23E-12
	BC-AC	-0.014	-0.015	-0.012	1.23E-12
	A-AC	-0.037	-0.039	-0.035	1.23E-12
	B-AC	-0.029	-0.031	-0.027	1.23E-12
	C-AC	-0.046	-0.047	-0.044	1.23E-12
	A-BC	-0.024	-0.025	-0.022	1.23E-12
	B-BC	-0.015	-0.017	-0.014	1.23E-12
	C-BC	-0.032	-0.034	-0.030	1.23E-12
	B-A	0.008	0.006	0.010	1.23E-12
	C-A	-0.008	-0.010	-0.007	1.23E-12
	C-B	-0.017	-0.018	-0.015	1.23E-12
demography	SGD-RGD	0.003	0.003	0.004	1.24E-12
locus:demography§	ABC:SGD-ABC:RGD	0.001	-0.002	0.004	0.96
	AB:SGD-AB:RGD	0.002	0.000	0.005	0.2
	AC:SGD-AC:RGD	0.001	-0.002	0.004	1
	BC:SGD-BC:RGD	0.002	-0.001	0.005	0.24
	A:SGD-A:RGD	0.007	0.004	0.010	2.54E-12
	B:SGD-B:RGD	0.007	0.004	0.010	1.39E-12
	C:SGD-C:RGD	0.001	-0.002	0.004	0.95

lwr: lower end point of the interval, upr: upper end point, p adj: p-value after adjustment for the multiple comparisons, RGD: rapid genetic drift, SGD: slow genetic drift.

§: only the RGD-SGD comparisons within each locus are shown.
