# Supplementary material for: HLA class I molecular variation and peptide-binding properties suggest a model of joint divergent asymmetric selection
Source: Immunogenetics. 2016 May 27;68(6):401–16. doi: 10.1007/s00251-016-0918-x (PMC4911380; doi:10.1007/s00251-016-0918-x)
Supplement: Supplementary file 3 — Supplementary Figs. S1 (a and b), S2, S3 (a and b), and S4 (a and b) (PDF 182 kb) [file 251_2016_918_MOESM3_ESM.pdf]

Supplementary Figures

Figures S1a and S1b

Density curves of the pairwise distances among 328 HLA class I alleles; (a) based on exons 2 and 3 sequence alignment (pairwise molecular distances, *PMD*) and (b) based on peptide binding prediction (pairwise peptide-binding distances, *PPBD*).

Figure S2

Number of alleles (k) and sample size (log transformed) in 46 human populations. The geographic provenance of each population is indicated by a colored dot. Populations are subdivided into RGD (rapid genetic drift, on the left plots) and SGD (slow genetic drift, on the right plots).

Figures S3a and S3b

Empirical distribution of the correlation coefficient (rsim) between k and (a) the mean pairwise molecular distance (mean *PMD*) or (b) the mean pairwise peptide-binding distance (mean *PPBD*) at each locus obtained through 2,500 random samplings (see material and methods). The dotted lines indicate the 2.5 and 97.5 percentile, respectively. The correlation coefficient observed at each locus is shown with a red line (see Table 3).

Figures S4a and S4b

Density curves of (a) the mean relative increase in molecular distance (*RIMD*) and (b) the mean relative gain in peptide binding coverage (*RGPBC*) in 46 human populations. Demography is indicated by colors (blue for small-sized and isolated populations

characterized by rapid genetic drift (RGD) and green for large outbred populations with expected slow genetic drift (SGD)).

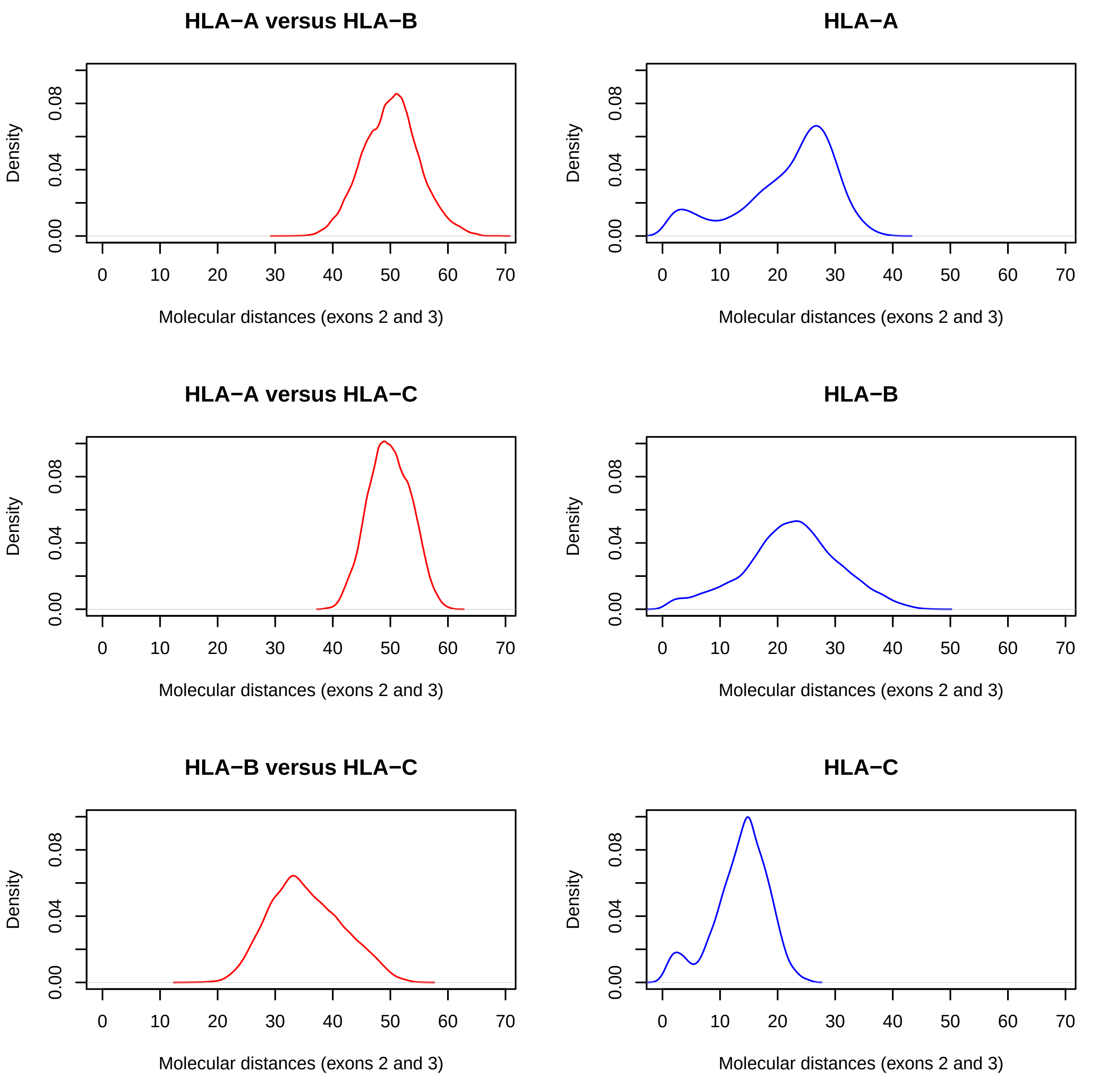
HLA−A versus HLA−B
HLA−A
HLA−A versus HLA−C
HLA−B
HLA−B versus HLA−C
HLA−C
Density
0.00
0.04
0.08
0
10
20
30
40
50
60
70
Molecular distances (exons 2 and 3)

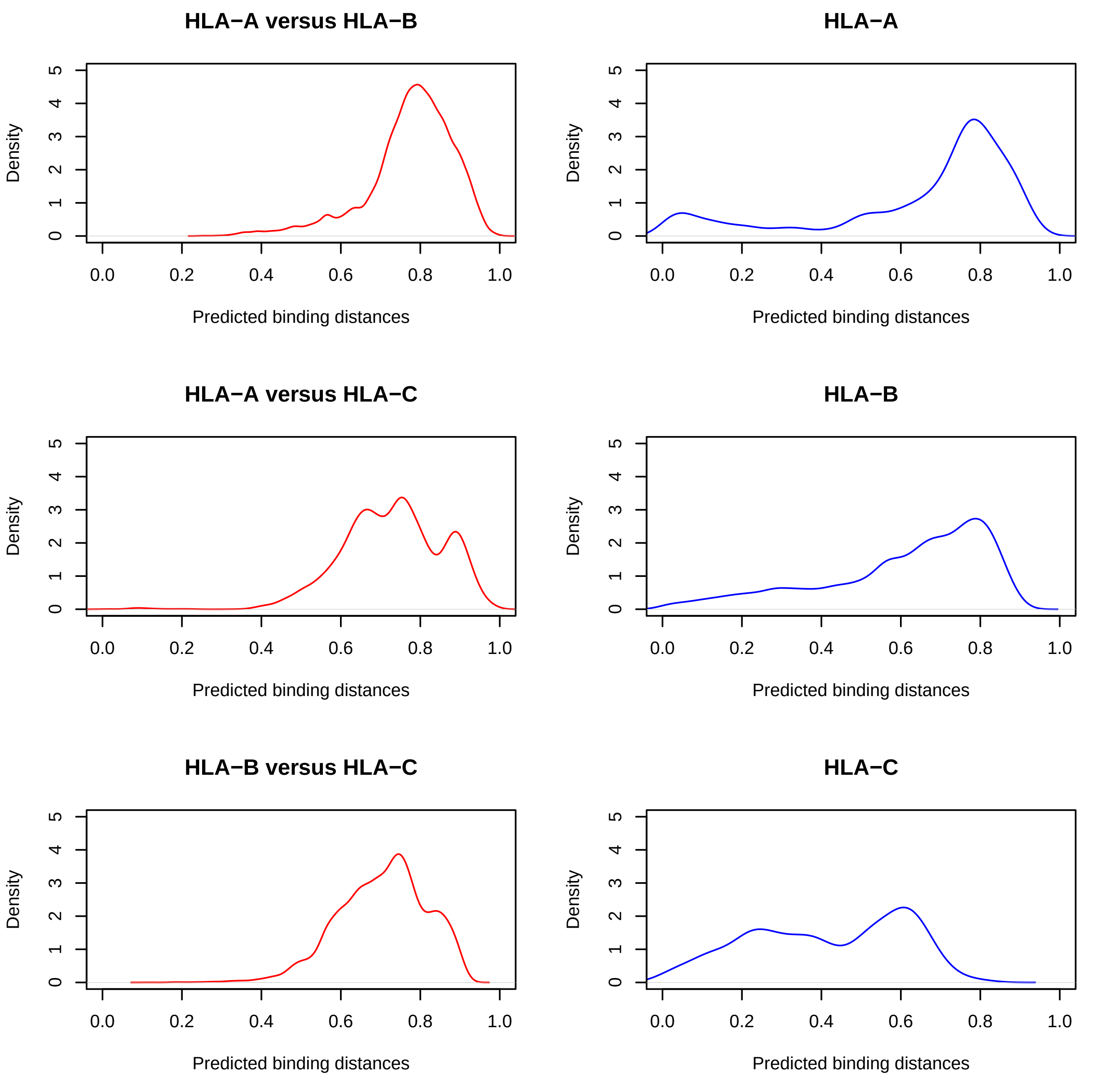

HLA−A versus HLA−B
HLA−A
HLA−A versus HLA−C
HLA−B
HLA−B versus HLA−C
HLA−C
Density
0 1 2 3 4 5
0.0 0.2 0.4 0.6 0.8 1.0
Predicted binding distances

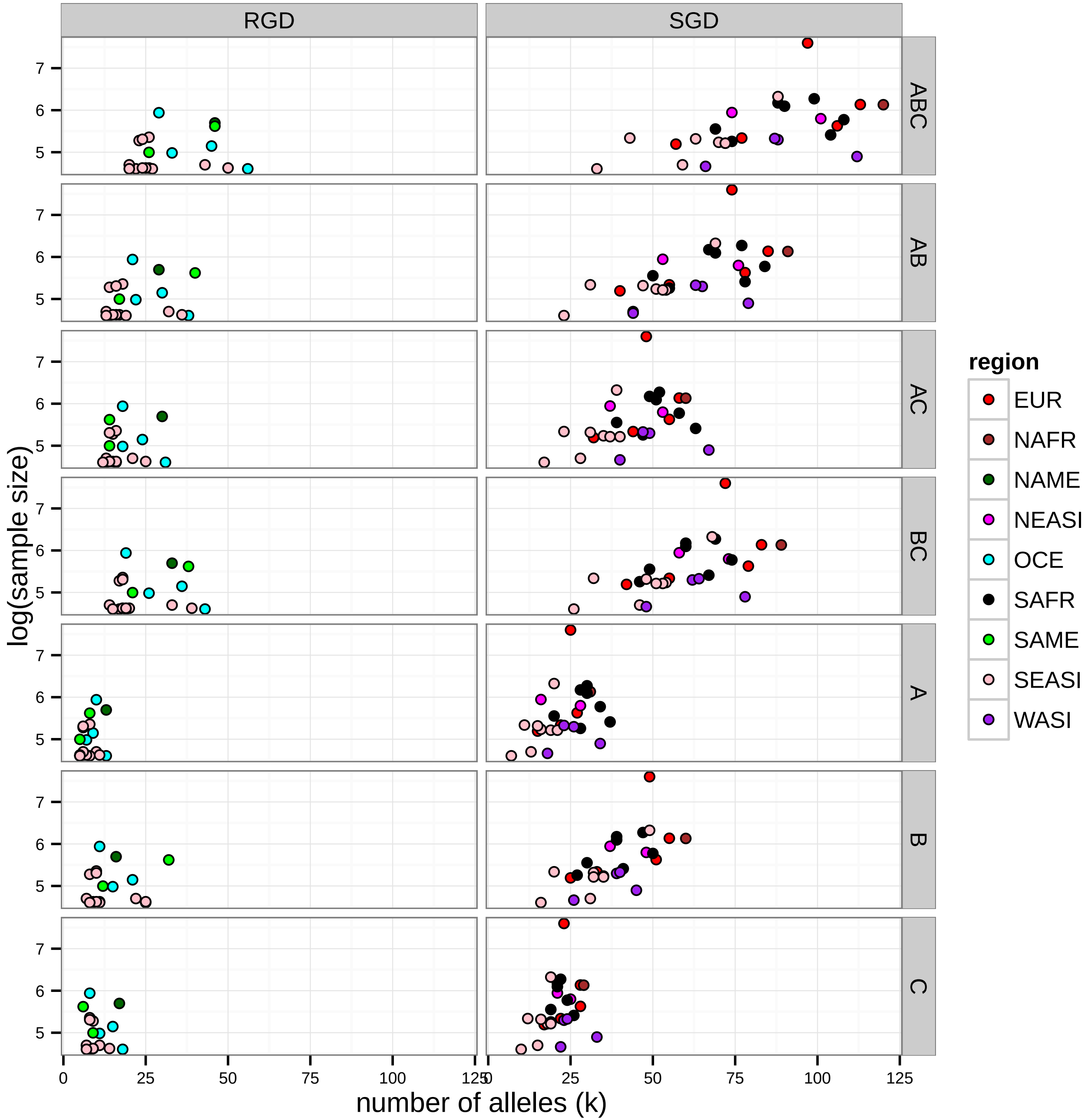

RGD
SGD
ABC
AB
AC
BC
A
B
C
log(sample size)
number of alleles (k)
region
EUR
NAFR
NAME
NEASI
OCE
SAFR
SAME
SEASI
WASI

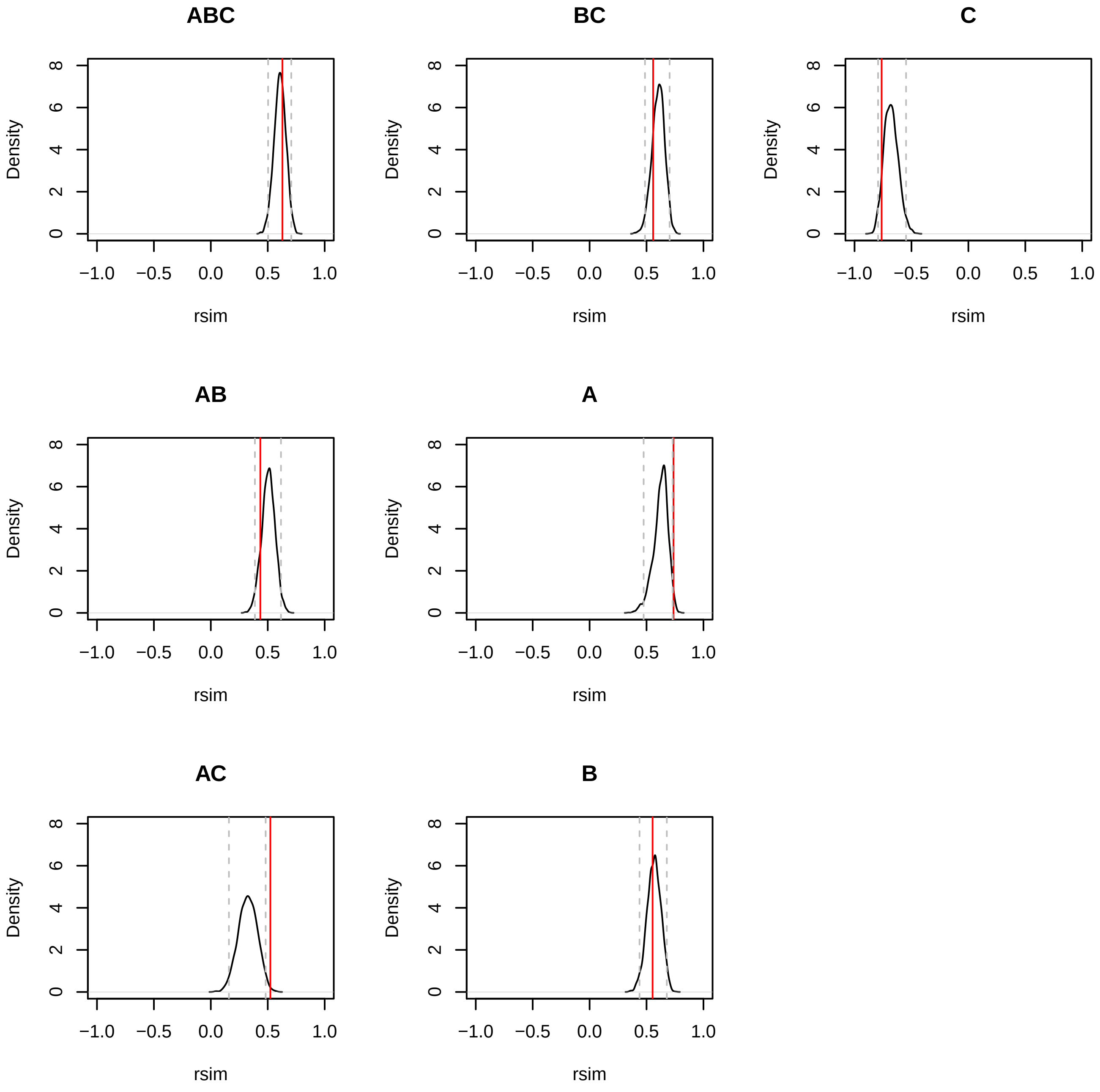

ABC
BC
C
AB
A
AC
B
Density
rsim
−1.0
−0.5
0.0
0.5
1.0
0
2
4
6
8

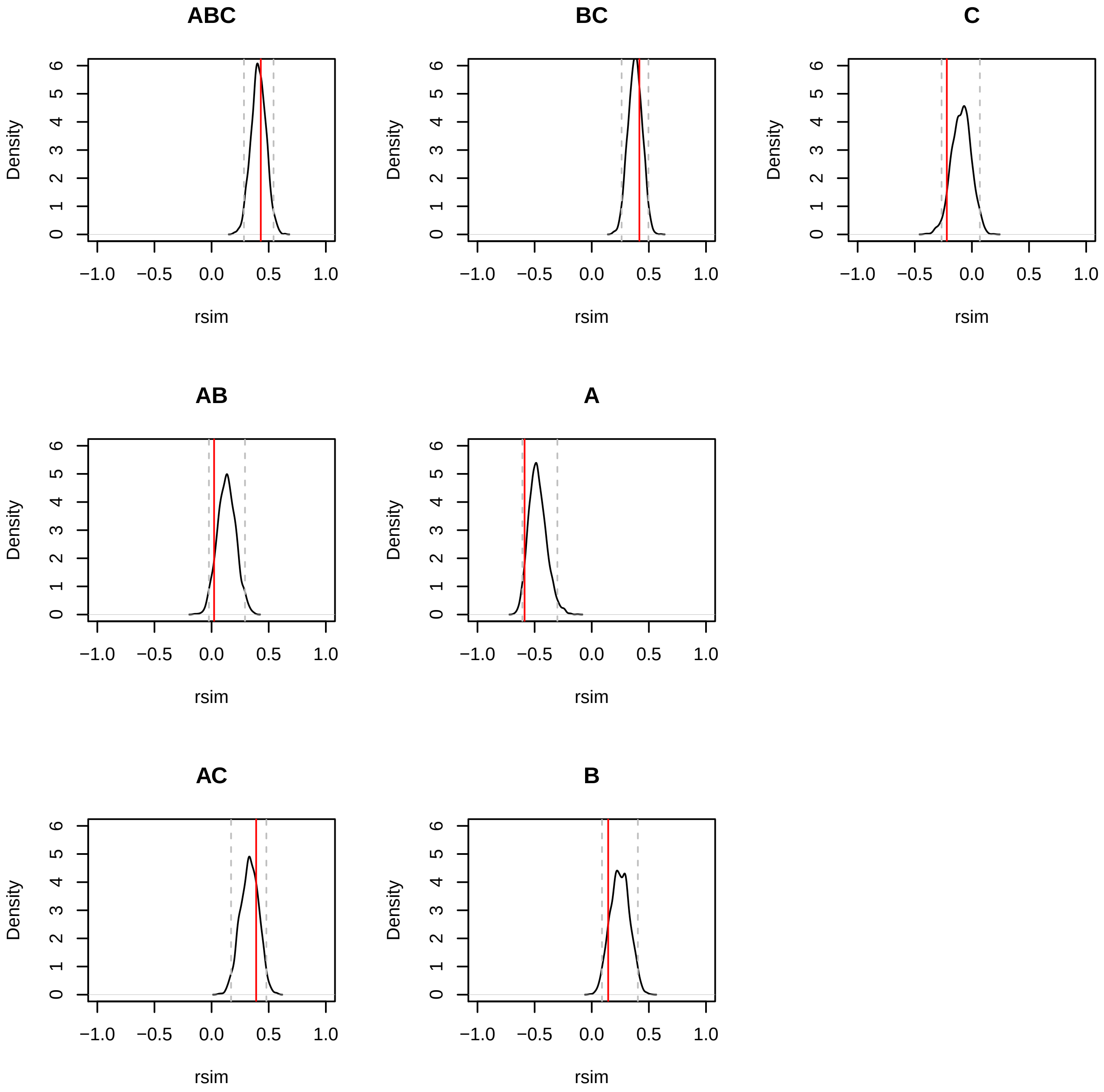
ABC
BC
C
AB
A
AC
B
Density
rsim

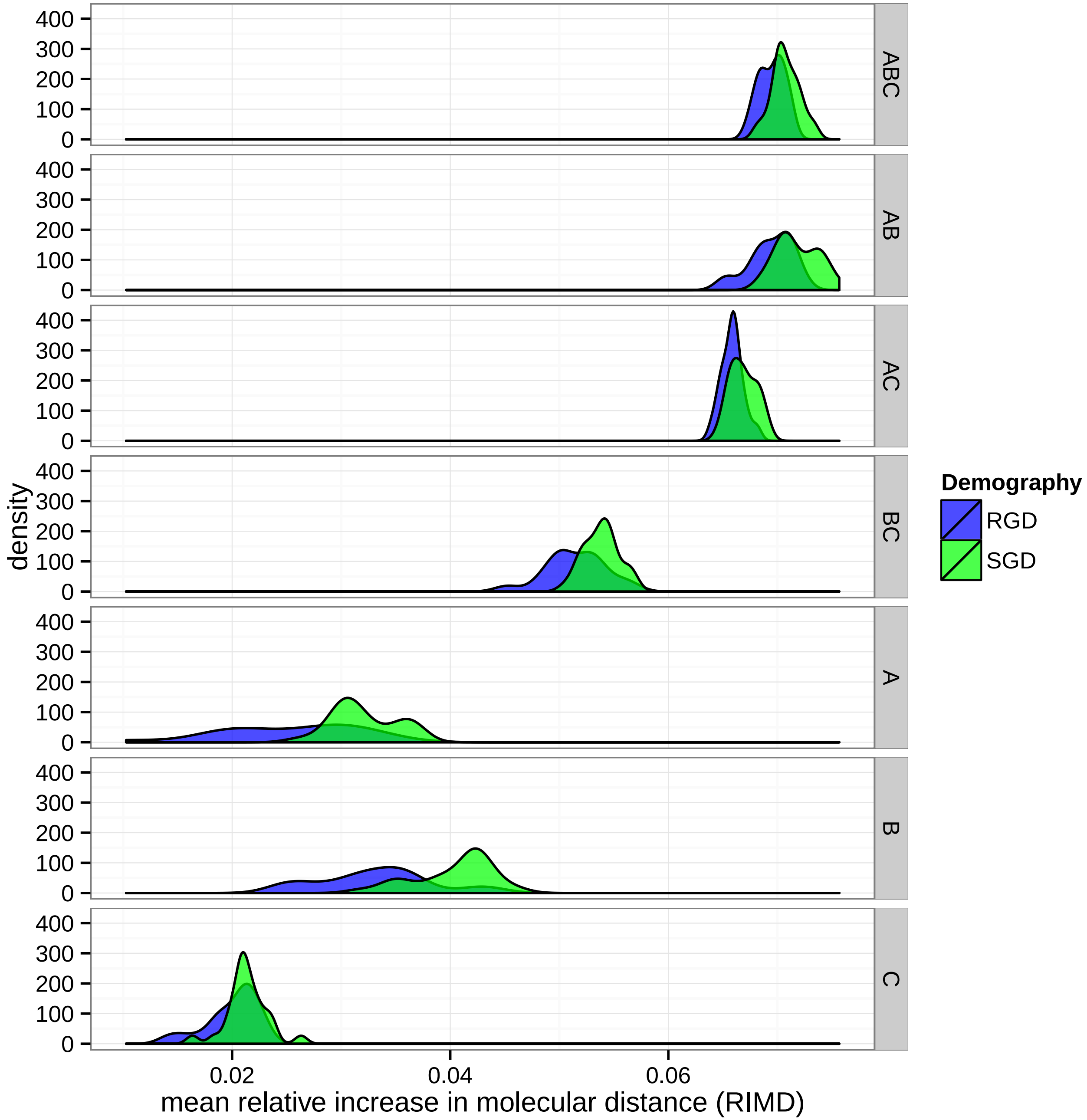

ABC
AB
AC
BC
A
B
C
400
300
200
100
0
density
0.02
0.04
0.06
mean relative increase in molecular distance (RIMD)
Demography
RGD
SGD

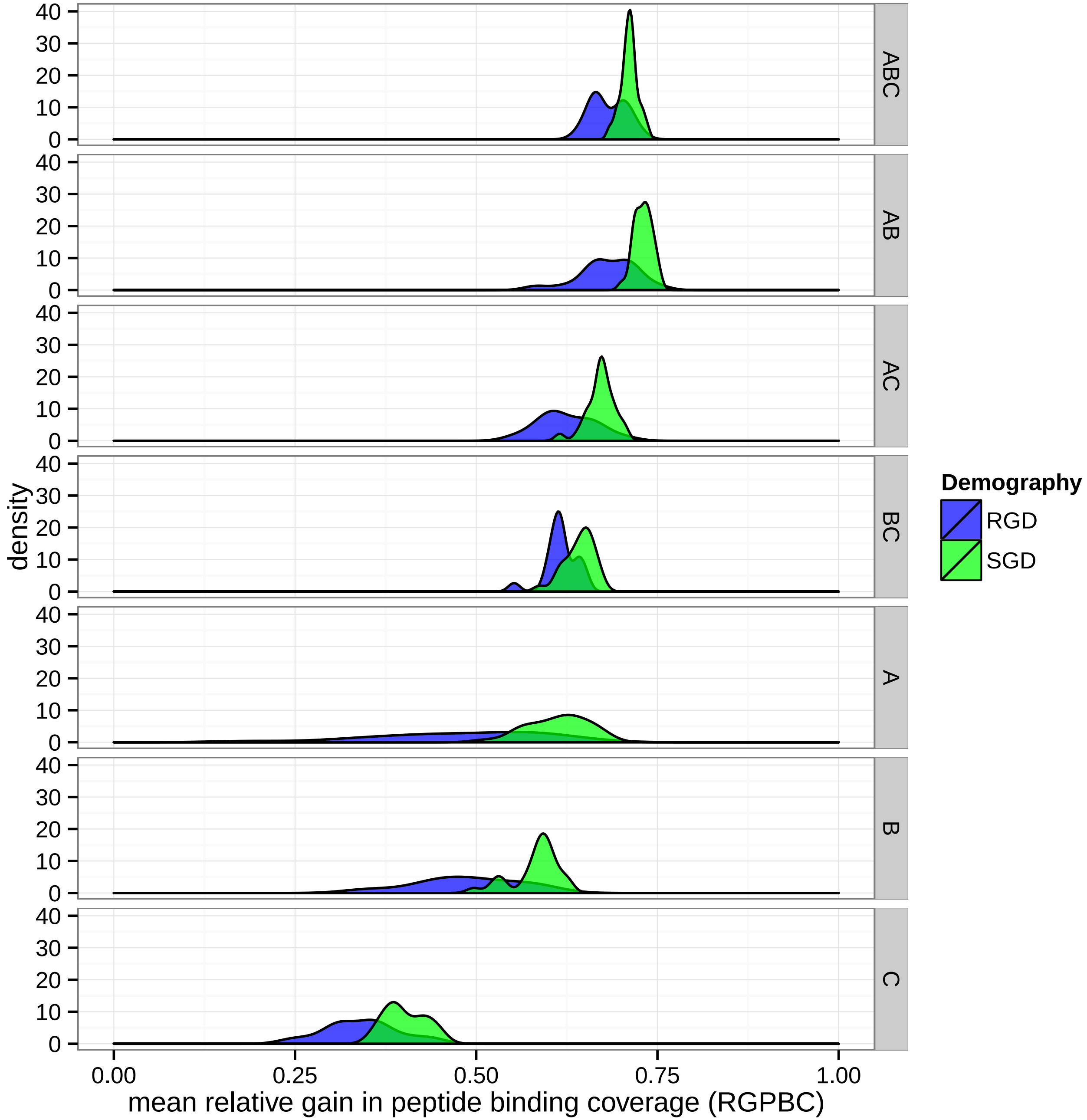

density
mean relative gain in peptide binding coverage (RGPBC)
0.00
0.25
0.50
0.75
1.00
0
10
20
30
40
ABC
AB
AC
BC
A
B
C
Demography
RGD
SGD
